# Large‐Area Conductor‐Loaded PDMS Flexible Composites for Wireless and Chipless Electromagnetic Multiplexed Temperature Sensors

**DOI:** 10.1002/advs.202412066

**Published:** 2025-01-28

**Authors:** Benjamin King, Nikolas Bruce, Mahmoud Wagih

**Affiliations:** ^1^ James Watt School of Engineering University of Glasgow Glasgow G12 8QQ UK

**Keywords:** electromagnetic sensing, passive electronics, polymer composites, RF sensors, temperature sensing

## Abstract

Capacitive dielectric temperature sensors based on polydimethylsiloxane (PDMS) loaded with 10 vol% of inexpensive, commercially‐available conductive fillers including copper, graphite, and milled carbon fiber (PDMS‐CF) powders are reported. The sensors are tested in the range of 20–110 °C and from 0.5 to 200 MHz, with enhanced sensitivity from 20 to 60 °C, and a relative response of 85.5% at 200 MHz for PDMS‐CF capacitors. PDMS‐CF capacitors are interrogated as a sensing element in wirelessly coupled chipless resonant coils tuned to 6.78 MHz with a response in the resonant frequency (*f*_r_) of the sensor, demonstrating an average sensitivity of 0.38% °C^−1^, a 40x improvement over a pristine PDMS capacitive sensor and outperforms state‐of‐the‐art frequency‐domain radio frequency temperature sensors. Exploiting its high sensitivity, the wireless sensing platform is interrogated using a low‐cost, portable, and open‐source NanoVNA demonstrating a relative response in *f*_r_ of 48.5%, good agreement with instrumentation‐grade vector network analyzers (VNAs) and negligible change in performance at a range of reading distances and humidities. Finally, a wireless tag is demonstrated with rapid, reversible dynamic response to changes in temperature, as well as the in the first scalable, multiplexed array of chipless sensors for spatial temperature detection.

## Graphical abstract

This work demonstrates the first chipless temperature sensing array based on conductor‐loaded composites. They allow multiple items to be simultaneously identified and monitored through multiplexed electromagnetic reflections. The work studies the influence of conductive fillers on the frequency‐domain properties of PDMS, for the first time. Using conductive composites as capacitive sensors yields rapid, robust, and spatial resonant frequency‐based readout.

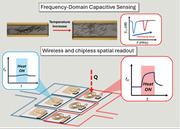

## 1 Introduction

With the rapid development of smart devices for body area networks and smart packaging, there is greater demand for flexible, lightweight, and conformable large‐area sensors which will enable more facile monitoring of physiological parameters and consumer products.^[^ ^1^, ^2^, ^3^ ^]^ Temperature is among the most fundamental measured physical parameters and reflects both the state of the object and the surrounding environment.^[^ ^4^, ^5^ ^]^ Measurement and control of temperature are important in many fields, including medicine, manufacturing, and agriculture, further demonstrating the demand for the development of inexpensive, large‐area sensors.

Functional polymer‐based composites have demonstrated excellent potential as sensors primarily owing to the combination of properties of the polymer (low density, ease of processing, inherent flexibility, and biocompatibility) and filler material (high thermal or electrical conductivity, excellent mechanical properties) being imparted to the resulting composite.^[^ ^6^, ^7^, ^8^ ^]^ Composites consisting of a polymer matrix loaded with conductive filler, in particular, have been widely reported as smart flexible strain sensors and piezoelectric sensors based on the variation of electrical resistance due to externally applied force,^[^ ^9^ ^]^ resulting from interactions of adjacent conductive filler particles or fibers.^[^ ^10^ ^]^ Conductor‐loaded polymer composites have also been reported as temperature sensors, including copolymer systems loaded with acid‐treated carbon black^[^ ^11^ ^]^ and nickel microparticle‐loaded polymer blends.^[^ ^12^ ^]^ Carbon nanomaterials including carbon nanotubes and graphene have also been widely studied in wearable electronics and temperature sensors owing to their excellent mechanical and chemical stability and high thermal conductivity.^[^ ^13^, ^14^, ^15^ ^]^

Sensors based on composites made from polydimethylsiloxane (PDMS) have excellent thermal stability, water and oxidation resistance, and biocompatibility.^[^ ^16^, ^17^ ^]^ Applications of PDMS‐loaded composites include stretchable conductive films for printed electronics,^[^ ^18^, ^19^ ^]^ and capacitive pressure sensors.^[^ ^20^, ^21^, ^22^ ^]^ PDMS composites loaded with carbon nanotubes (CNTs) have shown some temperature‐dependent response to strain, demonstrating that conductive PDMS composites have some temperature sensitivity.^[^ ^23^ ^]^ PDMS and conductive PDMS composites have been reported as temperature sensors, owing to their ability to undergo thermal expansion when heated.^[^ ^24^ ^]^ Loading PDMS with conductive materials is one strategy for enhancing its temperature sensitivity,^[^ ^25^ ^]^ since temperature fluctuation results in mechanical deformation of the polymer matrix, inducing separation of conductive networks and resulting in a change in the DC resistance or capacitance of the material.^[^ ^26^ ^]^ Conductive materials loaded into PDMS composites for enhancing their temperature sensitivity include graphite powder,^[^ ^27^, ^28^ ^]^ graphene,^[^ ^29^ ^]^ carbon nanotubes,^[^ ^30^ ^]^ and polyaniline.^[^ ^31^ ^]^

A key limitation of many state‐of‐the‐art polymer composite temperature sensors is that they are read as direct current (DC) sensors, meaning that they can only extract sensing measurements by measuring the DC resistance of the material. Alternating current (AC)‐based sensing and electromagnetic (EM) or radio frequency (RF) sensing enables the complex impedance response to be read, and can deliver improved sensitivity and linearity of response from the kHz to GHz‐frequency range.^[^ ^32^, ^33^, ^34^ ^]^ Additionally, these devices can be integrated into battery‐free, wirelessly powered, or passive systems, eliminating the need for an external power supply and making devices more compact.^[^ ^35^ ^]^ However, many wireless and battery‐free sensors still rely on the integration of chips for wireless readout, increasing the complexity of the system.^[^ ^36^, ^37^, ^38^ ^]^ EM‐based measurements have been demonstrated to enhance the performance of commercially available gas sensors^[^ ^32^ ^]^ and enabled a broadening of the range of temperature sensors from PDMS composites.^[^ ^33^ ^]^ However, enhancements in temperature sensing have only been demonstrated in the GHz part of the EM spectrum, with no investigation of frequency‐specific sensitivity enhancement.^[^ ^33^ ^]^ Additionally, a sensor with readout at GHz frequency, which detects sub‐MHz or dB changes in the resonant frequency or amplitude, respectively, requires precisely calibrated vector network analysis (VNA) hardware,^[^ ^39^, ^40^, ^41^, ^42^, ^43^ ^]^ limiting the practicality of these sensors and their ability to be read in varying and unknown environment outside of the laboratory, without a stable reference. Moreover, while passive chipless LC sensors have been explored for a variety of applications including biomedical sensors and consumer products,^[^ ^44^, ^45^, ^46^ ^]^ the low coupling between the reader and the sensor implies that specialized readout techniques are required,^[^ ^47^, ^48^ ^]^ with the observable resonance shift being limited to sub‐MHz levels.

**Figure 1:**
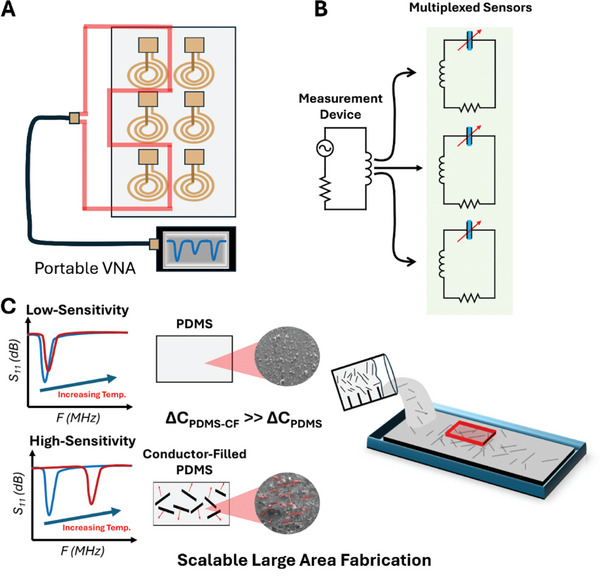
Principle of chipless multiplexed sensor platform based on conductor‐loaded PDMS composites. A) layout of 3 × 2 sensor array with reader coil connected to a portable VNA. B) Circuit diagram of RLC sensor tags with a variable capacitor. C) Sample response of sensor tags to temperature for PDMS (top) and PDMS‐CF (bottom), diagrams and inset of SEM images for PDMS and PDMS‐CF, and diagram of large‐area composite fabrication.

We present a chipless, multiplexed sensor array based on low‐cost wireless capacitive temperature sensors fabricated by loading a pure dielectric material (PDMS) with commercially available conductive fillers (**Figure** **1**A), investigating the readout sensitivity as a function of interfacing frequency, to enable highly sensitive and portable wireless and chipless electronics. These include graphite powder (90% < 44 µm diameter), milled carbon fiber (CF, 100 × 7.5 µm diameter), and copper powder (Cu, < 44 µm diameter) to fabricate a polymer matrix. The multiplexed sensors are based on coupled resistor‐inductor‐capacitor (*RLC*) circuits wirelessly coupled to a network analyzer (Figure 1B). Loading PDMS with conductive fillers results in a dielectric material with higher loss, and introduces a sensing mechanism based on the interaction of filler material, significantly enhancing the sensitivity of the device (Figure 1C, Left). The sensors were fabricated using a scalable molding process (Figure 1C, Right) and offer potential composite materials for the next generation of large‐area electronics.

## 2 Results and Discussion

### 2.1 Capacitive Properties of Loaded PDMS Composites

**Figure 2:**
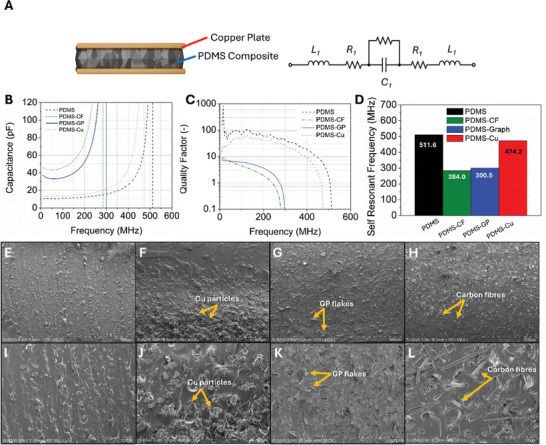
Schematic of conductor‐loaded dielectric and room‐temperature broadband composite‐based capacitor electrical properties. A) Schematic of PDMS composite capacitor and equivalent circuit model of capacitors in this work.^[^ ^53^ ^]^ B) capacitance as a function of frequency from 10 to 600 MHz for composite‐loaded capacitors, C) Quality factor (Q) at room temperature, and D) SRF of each capacitor. E–L) SEM images of loaded capacitors in this study with E–H) 100x magnification and I–L) 400x magnification. E,I) Pristine PDMS, F,J) PDMS‐Cu, G,K) PDMS‐GP, and H,L) PDMS‐CF.

The parallel plate capacitors (**Figure** **2**A) were characterized using a VNA up to 600 MHz to understand the influence of the composites’ loading material on its dielectric properties including capacitance (Figure 2B), quality factor (*Q*, Figure 2C), and self‐resonant frequency (SRF, Figure 2D). The PDMS‐CF and PDMS‐GP capacitors have a higher baseline capacitance compared to PDMS‐Cu and pristine PDMS capacitors, attributed to the larger number of conductive pathways that exist in the composite. To evaluate the losses in the loaded composites, *Q* up to 600 MHz was calculated based on the ratio of the imaginary part of the complex impedance (Im{*Z*}) and real part of the complex impedance (Re{*Z*}) using:

(1) Q=ImZRe{Z}

The quality factor of the carbon‐loaded composites PDMS‐CF and PDMS‐GP are six to eight‐fold lower than PDMS up to 200 MHz attributed to the conductive (lossy) fillers in the composite, increasing its loss tangent (tan δ  =  1/*Q*). The frequency‐dependent quality factor of the PDMS‐Cu capacitor follows a similar trend to that of pristine PDMS but is consistently lower in magnitude, owing to the Cu imparting some influence on the complex permittivity of the material and making the capacitor less ideal.^[^ ^49^ ^]^ PDMS‐CF and PDMS‐GP composite capacitors had a reduced *Q* compared to PDMS‐Cu and pristine PDMS capacitors, PDMS‐Cu had a relatively low average capacitance and elevated Q approaching the behavior of pristine PDMS devices, as opposed to the carbon conductor‐loaded composites. This can be attributed to the oxidation of the copper powder after exposure to air post‐manufacturing^[^ ^50^ ^]^ and the resulting presence of a mixture of semiconducting copper oxide and small quantities of conductive copper. It is important to note that beyond 200 MHz, the calculated capacitance of all four composites begins to increase exponentially with frequency, as the devices approach their SRF.^[^ ^51^, ^52^ ^]^

The SRF of each composite, at which Im {*Z*} =  0 Ω is heavily impacted by the incorporation of conductive fillers. The carbon‐loaded composites PDMS‐CF and PDMS‐GP have self‐resonant frequencies of 284.0 and 300.5 MHz, respectively, which are significantly lower than, pristine PDMS. The mechanism of decreasing SRF for carbon‐based fillers comes from loading dielectric materials with conductive (lossy) fillers, which decreases the ideality of the device by inducing parasitic inductance. The morphology of CF, which will be discussed shortly in more detail, has a larger aspect ratio (L/D) than GP which enhances the interactions of conductive networks in the composite, resulting in greater dielectric loss (lower Q), and a reduced SRF compared to PDMS‐GP.

To understand the distribution of the loaded material in the polymer composite matrix and develop relationships between their morphologies and electrical properties, scanning electron microscopy (SEM) images were taken of the conductive composites (Figure 2E–L). PDMS‐GP (Figure 2G,K) and PDMS‐CF (Figure 2H,L) composites have uniformly distributed and predominantly randomly oriented conductive filler throughout the polymer, indicating that they are well mixed. However, PDMS‐Cu (Figure 2F,J) composites have regions of high Cu powder concentration and low Cu concentration throughout the cross‐section of the composite, which is attributed to its higher density of 6.31 g cm^−^^3^, compared to 1.8 g·cm^−^^3^ of the milled CF and 2.6 g·cm^−3^ of graphite powder.

To evaluate the scalability of PDMS‐CF composite capacitors, we fabricated parallel plate capacitors with areas of 2, 4, and 6 cm^2^ and evaluated their area‐normalized capacitance up to 200 MHz (Figures S1 and S2, Supporting Information). The absolute capacitance values across the entire frequency range (Figure S1A, Supporting Information) increased linearly with increasing area, indicating a uniform permittivity laterally through the composite. The average area‐normalized capacitance is presented in Figure S2C (Supporting Information) and demonstrates that the capacitance of PDMS‐CF composites is in the range of 10–14 pF up to 200 MHz. Additionally, the standard deviation in capacitance up to 200 MHz is under 6.2% of the average value, demonstrating that CF‐loaded PDMS capacitors are well‐mixed over a large area and behave as parallel plate capacitors as the size of the device is changed.

### 2.2 Broadband Dielectric Sensor Performance

**Figure 3:**
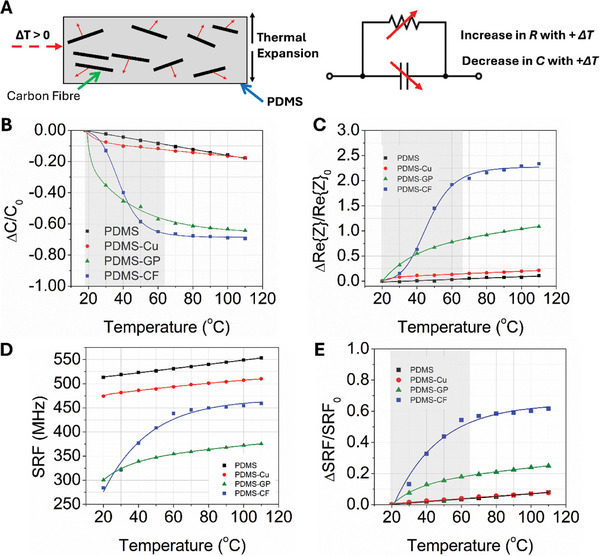
Temperature‐response data of conductor‐loaded composites in parallel plate capacitors. A) Sensing mechanism of conductor‐loaded PDMS composites with applied heat. B) Relative response to temperature in capacitance at 10 MHz. C) Relative response to temperature in real impedance (Re{Z}) at 10 MHz. D) Raw response to temperature in SRF. E) Relative response to temperature in SRF. The reference temperature for these measurements was taken at room temperature (20 °C) and the shaded region is the target sensing range. Measurements were taken for composite capacitors of pristine PDMS (black squares), PDMS‐Cu (red circles), PDMS‐GP (green triangles), and PDMS‐CF (blue squares).

The parallel plate capacitors were characterized for their temperature sensitivity by placing them on a heating mantle and heating them in increments of 10 °C in the range of 20–110 °C. In all cases, capacitance decreases as temperature increases. The relative response in capacitance versus temperature in the range of 0.5–200 MHz is shown for all four composites studied in this work in **Figure** **3**B with broadband data up to their SRF at 20, 40, 60, and 110 °C shown in Figure S4 (Supporting Information). The sensitivity of the four composites as temperature sensors is reported in Table S1 (Supporting Information). The inverse relationship between capacitance and temperature for PDMS and PDMS composites is attributed to a decrease in permittivity,^[^ ^54^ ^]^ and thermal expansion of the PDMS.^[^ ^55^ ^]^

The capacitance of pristine PDMS devices in this work (Figure 3B, black squares) changes linearly with temperature with an average sensitivity of 0.18%·°C^−1^ at all three investigated frequencies of 10 (Figure 3C), 100, and 200 MHz (Figure S6, Supporting Information). PDMS‐CF (blue squares) and PDMS‐GP (green triangles) show slightly different kinetics in response to temperature, with the response of PDMS‐CF capacitors saturating rapidly above 60 °C at a relative response of over 80% and PDMS‐GP following an exponential decay type response and starting to saturate above 90 °C. The temperature response of PDMS‐GP follows a similar trend to reported capacitors in the literature whose DC response followed exponential decay in the range of 30–110 °C for 15–25% graphite‐loaded composite dielectrics.^[^ ^27^ ^]^ For both PDMS‐CF and PDMS‐GP composites, we attribute the rapid initial relative response in capacitance to the thermal expansion of PDMS breaking of conductive pathways in the composite,^[^ ^56^ ^]^ resulting in a rapid decrease in capacitance approaching that of PDMS. It should be noted that CF undergoes some contraction upon heating due to its negative thermal expansion coefficient^[^ ^33^ ^]^; however, we assume that this effect is negligible relative to the thermal expansion of PDMS and results in additional separation between conductive fillers. Finally, PDMS‐Cu capacitors (red circles) also demonstrated a nonlinear response at the three investigated frequencies, which indicates that while some oxidation of the copper particles likely occurred after manufacturing due to the exposure of particle surfaces to air, some conductive non‐oxidized copper is present in the composite.

The real impedance (Re{Z}) of each composite was measured as a function of temperature from 10 to 200 MHz and in the range of 20–110 °C (Figure S5, Supporting Information) to represent the resistive component of each composite sensor, with the relative response to temperature at 10 MHz shown in Figure 3C. Across the range of composites, PDMS‐CF has the lowest Re{Z} at room temperature and the most significant relative response at frequencies approaching DC. However, pristine PDMS also demonstrates a significant reduction in Re{Z} with increasing frequency, which is consistent with AC measurements in the literature.^[^ ^57^ ^]^ While the relative response in Re{Z} is larger than in capacitance (Im{Z}) for each composite, capacitive sensing presents inherent advantages including low hysteresis and power consumption compared to resistive sensors.^[^ ^58^ ^]^ Critically, capacitive RF sensors can be operated by reading changes in resonant frequency,^[^ ^59^ ^]^ while resistive sensors are only read out by measuring the amplitude of the resonant frequency and therefore need to be calibrated for each desired discrete reading distance.^[^ ^60^ ^]^

Remarkably, the loaded composites did demonstrate a frequency‐dependent response to temperature, with operating PDMS‐CF at 200 MHz resulting in an enhancement of 25% at 50 °C and 15% above 60 °C. Additionally, PDMS‐GP consistently demonstrated a 3–5% enhancement in relative response operating at 10 MHz compared to 100 MHz. Unlike previous studies on PDMS‐based capacitors operating at DC, this work demonstrates that an optimum operating frequency exists for capacitive temperature sensors, which has been explored in capacitive humidity sensors in the kHz range,^[^ ^61^ ^]^ but has not previously been reported for PDMS composite‐based temperature sensing materials. This temperature‐sensing approach demonstrates the limitations of DC sensing and provides further motivation for exploring the frequency dependence of sensing materials.

In addition to the frequency‐dependent response to temperature, pristine PDMS and PDMS composites demonstrate a positive shift in SRF as a function of temperature, shown in Figure 3E and Figure S4 (Supporting Information). PDMS and PDMS‐Cu demonstrate a similar linear increase in SRF of 0.444 and 0.400 MHz·°C^−1^, respectively, while PDMS‐CF and PDMS‐GP undergo an initial rapid increase of 3.850 and 1.350 MHz·°C^−1^ between 20 and 60 °C, followed by an increase of 0.420 MHz·°C^−1^ for both composites from 60 to 110 °C which is similar to pristine PDMS. These results suggest that the initial rapid increase in SRF of GP and CF‐loaded composites is attributed to the separation of lossy filler, while beyond 60 °C the temperature response is primarily attributed to the thermal expansion of PDMS. However, PDMS‐Cu, which is loaded with a lower‐conductivity filler, behaves similarly to pristine PDMS.

### 2.3 Wireless and Chipless Temperature Sensing

**Figure 4:**
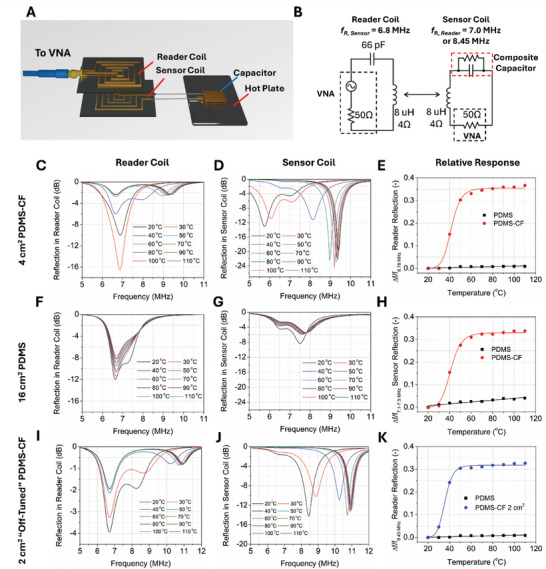
Chipless wireless readout using a tuned reader coil in the same band (C–H) and off‐tuned reader coil (I–K): A) 3D render of wireless temperature sensing experiment. B) Equivalent circuit of coupled RLC coils. C–E) reflection coefficient (S_11_) data and relative response in reader coil for tuned 4 cm^2^ PDMS‐CF capacitor. F–H) reflection coefficient (S_11_) data and relative response in reader coil for 16 cm^2^ tuned PDMS capacitor. I–K) Reflection coefficient (S_11_) data and relative response in reader coil for detuned 2 cm^2^ PDMS‐CF capacitor with a reference resonant frequency of 8.45 MHz.

To demonstrate the applicability of conductive composite temperature sensors as passive measurement devices, we fabricated a sensing system based on coupled RLC coils and took sensing measurements in the temperature range of 20–110 °C (**Figure** **4**A). To enable wireless temperature readout, the top coil (“Reader”) was tuned to 6.78 MHz, the standard international, scientific, and medical (ISM) band, and is an attractive frequency of operation from a regulatory‐compliance and standardization perspective, widely used in inductive power transfer applications.^[^ ^52^ ^]^ Pristine PDMS capacitors and PDMS‐CF capacitors were sized and connected to a separate RLC coil (“Sensor”) to be tuned to the 6.78 MHz band. The reader and sensor coils were spaced by 45 mm, which achieves critical inductive coupling without observing multiple resonances (frequency splitting), which is common at small separation distances, due to over‐coupling.^[^ ^62^ ^]^ The simplified equivalent circuit diagram of the coupled coils is shown in Figure 4B. Changes in the reflection coefficient of the reader coils for tuned wireless PDMS‐CF and PDMS systems are shown in Figure 4C,F, while changes in the reflection coefficient of sensor coils are shown in Figure 4D,G, respectively. In the wireless measurement of each capacitor, changes in the resonant frequencies of the sensor and reader coils are observed and quantified in Figure 4E,H.

Observable at both the sensor and reader coils, the PDMS‐CF‐based sensor demonstrates a significant positive shift in the resonant frequency, which is attributed to the previously observed decrease in capacitance of the sensing material.^[^ ^63^ ^]^ The change in the resonant frequency of the sensor coil results in the coils being poorly matched at 6.78 MHz and changes in both the amplitude, at resonance. Furthermore, an additional resonance is created by the changing capacitance detuning the sensor coil, creating a readable sensor response through Δ*f*_r_. This change in the resonant frequency can be quantified as the temperature response of the wireless system and has similarly been applied to measure various measurands including ice accumulation^[^ ^64^ ^]^ and strain sensing.^[^ ^65^ ^]^

In the reader coil and for the PDMS‐CF sensor, the resonant frequency increased by 36%, from 6.88 to 9.41 MHz, compared to 0.9% for the pristine PDMS capacitor showing an increase from 6.63 to 6.69 MHz. In the sensor coil itself, where the capacitor uses the PDMS‐CF dielectric, the resonant frequency increased by 33.8% from 7.03 to 9.41 MHz compared to 4.10% for PDMS whose resonant frequency increased from 7.50 to 7.81 MHz.

While the frequency response of 4 cm^2^ PDMS‐CF wireless sensors significantly outperformed pristine PDMS wireless sensors, the resonant frequency change was negligible between 20 and 30 °C and only an increase in the amplitude of the reflection was observed, increasing from −10.0 to −16.5 dB. This is attributed to the very small change in permittivity, predominantly influencing *f*_r_, compared to the change in the conductivity, which influences the equivalent series resistance and hence the losses in the capacitor. As such, the increase in reflection can be explained by occurred due to improved matching between the coils at 6.88 MHz as temperature increased from 20 to 30 °C, due to the improved matching between the source and 50 Ω termination on the sensing LC resonator. As the temperature rises further, the sensor coil is detuned, resulting in higher reflections at the unloaded resonant frequency.

This amplitude increase without resonant frequency yields a temperature sensor that is only reliable above 30 °C. To address this, we adopted a novel readout mechanism where the sensor and reader coil are deliberately off‐tuned, to isolate these two opposing effects. This was achieved by fabricating an identical wireless sensor system with a PDMS‐CF capacitor having an area of 2 cm^2^ (≈23 pF), which resulted in a shift of the resonant frequency of the sensor coil from 7.03 to 8.45 MHz. The reflection of the reader coil of the system is shown in Figure 4I and the reflection of the sensor coil is shown in Figure 4J. The shift in the resonant frequency of the sensor coil is measurable by the reader coil, which shows a distinct second resonance peak at 8.26 MHz. By changing the area of the PDMS‐CF capacitor, the response in the resonant frequency of both the sensor and reader coils can be read in the full temperature range of 20–110 °C and the relative response of the reader coil is compared to the pristine PDMS system in Figure 4K. Similar to the wireless system with the integrated 4 cm^2^ PDMS‐CF capacitor, the relative response of the 8.26 MHz resonance of the reader coil shows a positive shift with temperature to 10.96 MHz, which is a 32.7% change, and still significantly outperforms the wireless PDMS sensing system by over thirty six‐fold. Additionally, deliberately de‐tuning the LC resonators by adjusting the size of the capacitor enabled an increase in the sensing range of the reader coil while only sacrificing a negligible magnitude of the relative response read by the better‐matched system with a 4cm^2^ PDMS‐CF capacitor. While wireless temperature sensing measurements for both PDMS‐CF coils were taken in the range of 20–110 °C, the optimum sensitivity of the wireless system occurs in the range of 20–60 °C, with a reduced sensitivity at high temperatures. This is attributed to the conductive system approaching the behavior of PDMS at high temperatures where the change in permittivity is dominated by thermal expansion of the PDMS rather than interactions between particles of conductive filler. Insets of broadband sensor response at high temperature are shown in Figure S6 (Supporting Information).

### 2.4 Wireless Sensor Readout Using a Portable Low‐Cost NanoVNA

**Figure 5:**
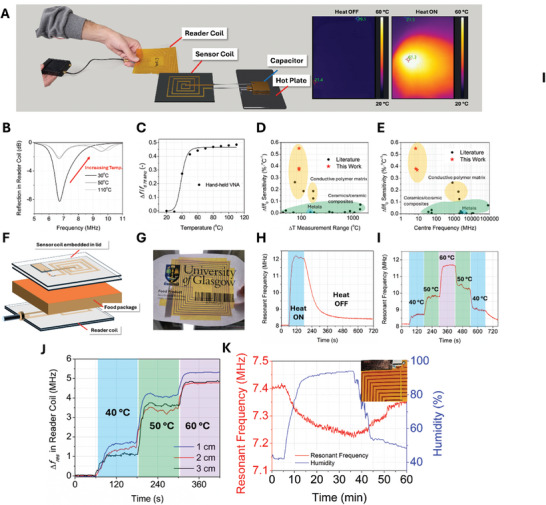
Chipless readout using inexpensive handheld VNA and practical packaging use‐case. A) Wireless sensing setup with NanoVNA connected to inductive coils and PDMS‐CF parallel plate capacitor and thermal camera images before and after heating the sensor tag. B) Change in reader coil reflection as a function of temperature for PDMS‐CF capacitor measured with the PicoVNA (solid lines) and NanoVNA (dashed lines). C) Relative response in the resonant frequency of the reader coil as a function of temperature measured with low‐cost handheld NanoVNA. D,E) Comparison with state‐of‐the‐art frequency‐domain temperature sensors in temperature measurement range and reference frequency (see Table S2, Supporting Information for the data and references). F) Schematic of smart packaging sensor system. G) Photo of sensor tag integrated into package lid. H) Dynamic response of sensor tag in *f*_r_ of the RL reader coil to step change in temperature relative to 20 °C. I) Dynamic response of sensor tag of *f*_r_ in the RL reader coil to 10 °C increments in temperature from 20 and 60 °C. J) Total change in *f*_r_ of the sensor tag at coupling distances of 1–3 cm. K) Humidity response in *f*_r_ of sensor tag with inset image of sensor tag at the maximum recorded humidity of 93.5%.

To validate the wireless temperature sensing capability of the PDMS‐CF composites under more practical test conditions, the sensor coil was measured using a lightweight, low‐cost, and portable VNA, the open source NanoVNA,^[^ ^66^ ^]^ (costing under $100, and occupying a smaller footprint than a mobile phone) which can measure reflection coefficients in the range of 50 kHz–1500 MHz. The temperature sensing setup with the NanoVNA is illustrated in **Figure** **5**A and the reflection coefficient at 30, 50, and 110 °C is shown in Figure 5B with the full temperature‐dependent reflection and transmission coefficient data sets for the NanoVNA in Figures S11 and S13 (Supporting Information). The temperature sensing readout by the calibrated bench VNA and NanoVNA are visually similar across the full temperature range (Figure S13, Supporting Information), with both sets of traces showing the evolution of a second peak beyond 8 MHz as temperature increases. However, the NanoVNA readout shows a greater shift in the reflection coefficient than the PicoVNA readout at each temperature, which results in an increase in the observed relative response in temperature Figure 5C. The broadband reflection coefficient response is similar between the two measurement setups, suggesting a good agreement between the portable and low‐cost readout, and the standard VNA used in most studies.^[^ ^39^, ^40^, ^41^, ^42^, ^43^ ^]^ The variations observed in the absolute change in the resonant frequency can be attributed to changes in the assembly of the measurement setup, where the separation and alignment between the inductively coupled coils.

These results demonstrate that, owing to its high sensitivity (Δ*C*,  Δtanδ) the PDMS‐CF capacitive sensors can be integrated into portable, low‐cost, and chipless wireless temperature detection systems. The achieved sensitivity, compared with wireless temperature sensors from the literature in Figure 5D,E, and Table S2 (Supporting Information), demonstrates how the combination of engineered composites and sensitivity‐driven readout frequency selection could achieve highly sensitive temperature detectors.

The average sensitivity of 0.55%·°C^−1^ over the range of 20–110 °C for the handheld VNA system outperforms state‐of‐the‐art RF temperature sensors, including resonator‐based RF temperature sensors based on CF‐loaded capacitors with loadings of up to 40% CF by weight.^[^ ^33^ ^]^ These previous sensors were reported based on engineered composites with comparable sensitivity (over 20% lower), however, the readout was only demonstrated at GHz frequencies, requiring more complex readout electronics, with no report of a chipless readout. Thus, the achieved sensitivity coupled with the demonstration of a chipless readout using a low‐cost/open‐source circuit creates a pathway to the widespread adoption of chipless frequency‐domain sensors outside of a laboratory or without access to expensive specialty equipment.

Motivated by the excellent response of the chipless sensor with the hand‐held VNA, we integrated the *RLC* coupled coil with a PDMS‐CF capacitor into a smart packaging application targeted toward food quality monitoring. Smart packaging is designed to interact with products in real time and can provide crucial data for food safety and security based on changes in temperature, pH change, microbial content, and oxidation.^[^ ^67^ ^]^ Monitoring the environmental conditions in which food is stored, including temperature, is critical for preventing spoilage and foodborne illness.^[^ ^68^ ^]^

We fabricated an additional coupled coil circuit by integrating a 4 cm^2^ PDMS‐CF capacitor into the footprint of the sensor coil and replacing the 50 Ω load of the hand‐held VNA with a 47 Ω resistor in series with the sensor coil and capacitor (Figure S17, Supporting Information). The sensor coil was then affixed to a paper sheet with polyimide tape and used as the lid of a simulated food container to act as a passive temperature sensor for food product quality control, with the reader coil at the base of the container (Figure 5G). To evaluate the dynamic response of the smart packaging tag, the container was heated by a stream of hot air at 60 °C on the bench top and sampled every 1.5 s (Figure 5H). The time constant (τ), which is the time taken for a sensor to reach 63.2% of its final value from exposure to a step change in temperature, was 14 s and a response of ≈0.1 MHz·s^−1^ from the start of heating until the sensor reaches steady state (≈40 s). While removing the capacitor from the reader coil resulted in a decrease in coupling (from −8 to ≈ −3 dB) coupling was sufficient to read the response in the resonant frequency, and the broadband data for both the step‐change response and the incremental response is shown in Figure S18 (Supporting Information). We then investigated the response of the sensor tag to incremental increases in temperature (Figure 5I) up to 60 °C and demonstrated a rapid, stepwise, and reversible response in resonant frequency with temperature. We also investigate the influence of coupling distance on the sensitivity of the tags to step changes in temperature, shown in Figure 5J. While the coupling distance has an influence on the resonant frequency of the reader coil, we demonstrate that the dynamic response in resonant frequency as a function of temperature is agnostic of coupling distance.

### 2.5 Response of Chipless Tag to Humidity

Humidity is a common environmental variable that can interfere with the performance of sensors by introducing charge traps in resistive sensors, or by altering the relative permittivity of the material in dielectric sensors.^[^ ^69^ ^]^ Therefore, we investigate the dynamic response of PDMS‐CF chipless tags to humidity in the range of 43.5–93.5% humidity with a separation distance between the sensor and reader coil of 2 cm (Figure 5K). The resonant frequency of the chipless tag decreased with increasing humidity by 0.2 MHz, representing a sensitivity of 0.004 MHz·% RH^−1^ compared to 0.11 MHz·°C^−1^ response of the tag in the range of 20–60 °C, demonstrating that the chipless tag is 28‐fold more sensitive to temperature than humidity.

### 2.6 Development of Chipless Tag Array for Scalable, Spatial Temperature Sensing

**Figure 6:**
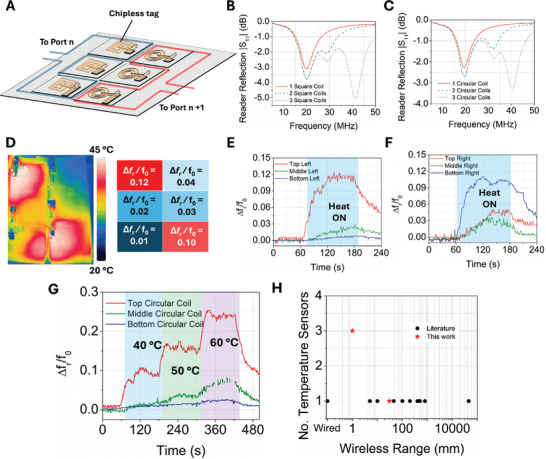
Spatial temperature detection with a wireless, chipless, and multiplexed array of RLC sensor tags. A) Schematic of six‐coil temperature array. B) Reflection coefficient (S_11_) of reader coil with sequential addition of circular sensor tags of increasing resonant frequency. C) Reflection coefficient (S_11_) of reader coil with sequential addition of square sensor tags of increasing resonant frequency. D) Thermal camera image showing the temperature of tag array with two tags simultaneously heated and heat map of sensor tags. E) Relative response in the resonant frequency of column of circular tags to temperature from the heating top left corner of the sensor array. F) Relative response in the resonant frequency of column of square tags to temperature from the heating bottom right corner of the sensor array, G) Relative response in resonant frequency to increments in temperature of 3 × 1 array of circular tags. H) Comparison to literature of number of sensors and reading distance for other AC/RF temperature sensors.

To demonstrate the scalability of the chipless sensing platform and expand their potential range of application, we fabricated an array of chipless *RLC* circuits with PDMS‐CF capacitors having areas of 0.7 cm^2^ × 4 cm^2^ and inductive coils having a diameter or side length of 5 cm for spatial temperature sensing (**Figure** **6**A; Figure S19, Supporting Information). The properties of the small sensing coils including Q, resistance, and inductance are provided in Figure S20 (Supporting Information). The tags resonate in the range of 20–45 MHz with a spacing of ≈12 MHz to provide sufficient bandwidth for response to changes in temperature without overlapping. The influence of adding additional sensor coils to the same reader on the reflection coefficient of the reader coil (*S*_11_) is shown in Figure 6B for a 3 × 1 array of square coils and Figure 6C for a 3 × 1 array of circular coils, with the entire system comprising a 3 × 2 array and two reader coils. The sequential addition of sensing tags to the reader coil demonstrates that while the amplitude of the resonant frequency changes slightly, the additional tags do not influence its position, meaning that the system can be read out as a frequency‐domain sensor array. Each reader coil accommodates up to three sensor tags on one port, meaning that the system can be scaled to accommodate 3*n* coils where *n* is the number of available ports, with the spatial resolution of the system being dictated by the footprint of the chipless tags. It should be noted that while the sensing array is fabricated to operate with two ports, the system can be scaled up with one reader and a switch to sequentially measure groups of tags. Without any tags, there is negligible influence of the proximity of the readers on the reflection coefficients (*S*_11_ and *S*_22_, Figure S21, Supporting Information).

The heat map of the chipless array (Figure 6D) demonstrates that heating different sections of the array simultaneously results in a temperature gradient across the tag that can be read wirelessly in the various resonant frequencies of the array elements. For both the column of tags with circular coils (Figure 6E) and square coils (Figure 6F), there is a clear relative change in resonant frequency from heating the array, with the most substantial change occurring where heat is directly applied and other tags reading changes in temperature from residual heat owing to heating being performed with a stream of hot air which propagates heat across the surfaces of the tags. The arrays of sensing tags are also sensitive to step changes in temperature as shown for the 3 × 1 array of circular tags in Figure 6G. The tag exposed directly to temperature undergoes a similar stepped response to the large tag in Figure 5J, while the two tags further from the heat source also demonstrate a progressively less strong relative response in resonant frequency related to their larger distance from the heat source (5 cm for the middle tag and 10 cm for the bottom tag, respectively).

While spatial and temporal mapping of temperature has been reported for up to 65 chipped sensors,^[^ ^70^ ^]^ our platform represents the first‐ever report of a chipless and scalable array for temperature mapping based on conductor‐loaded composites and demonstrates both wireless and spatial temperature sensing capabilities. A comparison of reading distance and number of sensors compared to state‐of‐the‐art AC/RF temperature sensors is shown in Figure 5H and demonstrates that our scalable system incorporates a greater number of readable sensors among literature examples with a reading distance of >0 m.

## 3 Conclusion

We presented the first holistic investigation combining composite formulation, readout frequency optimization, and a fully chipless demonstration of electromagnetic frequency‐based temperature sensing. The influence of a variety of inexpensive and commercially available carbon and metallic powders as filler materials in PDMS composites for capacitive‐based temperature sensors was explored. Loading PDMS with 10 vol% filler resulted in an increase in average room temperature capacitance from 3 pF for Cu powder (PDMS‐Cu) to 25–35 pF for graphite powder (PDMS‐GP) and milled carbon fiber powder (PDMS‐CF). PDMS‐GP and PDMS‐CF demonstrated a remarkable enhancement in temperature sensitivity compared to pristine PDMS with a relative response in capacitance of up to 67.1% and 85.5%, respectively, at 200 MHz. Additionally, we demonstrated that the operating frequency of the capacitors is highly influential in the sensing response of the devices, resulting in a change in relative response of up to 15.5% in capacitance between 10 and 200 MHz for PDMS‐CF devices.

The overall system was demonstrated as a passive, wireless capacitor by integrating it into a system of coupled *RLC* coils tuned to 6.78 MHz, demonstrating its response in resonant frequency compared to a PDMS sensor tuned to the same room temperature resonant frequency. The resulting wireless PDMS‐CF capacitor yielded a relative response in the resonant frequency of the reader coil of 33.8%, compared to 0.9% for PDMS, which is a 40x improvement in sensitivity. Using a novel frequency‐shifted readout, a reliable temperature readout between 20 and 110 °C with a negligible change in relative response characteristics was achieved, improving the robustness beyond state‐of‐the‐art passive LC sensors. The demonstrated sensitivity enabled the sensor to be interrogated using a low‐cost and open‐source VNA (the nanoVNA), in good agreement with calibrated instrumentation‐grade lab equipment. Using the low‐cost VNA and our PDMS‐CF formulation, we fabricated a smart packaging tag and recorded its dynamic response between 20 and 60 °C. The sensor tag had a time constant of 14 s and demonstrated a reversible response to incremental increases and decreases in temperature. These results demonstrate how large‐area sensors based on scalable and low‐cost processes could be sampled remotely without specialty equipment which could be adopted for widespread use. Finally, to demonstrate the scalability of the sensing platform, we fabricated the first example of chipless and wireless temperature sensors by tuning *RLC* circuits to resonate at frequencies between 20 and 45 MHz and demonstrated a 3 × 2 multiplexed array that was able to spatially detect temperature, using two readout coils, showing a promising approach for scaling electromagnetically multiplexed sensors.

## 4 Experimental Section

### Materials

Milled Carbon Fiber powder (D = 7.5 µm, L = 100 µm, FP‐MCF‐004), Graphite Powder (FP‐GP‐035), and Copper Metal Powder (325 mesh, FP‐COP‐025) were purchased from Easy Composites. SYLGARD 184 Silicone Elastomer Kit including polydimethylsiloxane (PDMS) and curing agent was purchased from Farnell.

### Fabrication of Loaded Composite Parallel Plate Capacitors

A summary of the mass and volume fractions of filler in each loaded composite in this study is shown in **Table** **1**. The requisite mass fraction of PDMS and filler is loaded into a beaker and stirred with a metal spatula until combined and well mixed. Next, the curing agent is added in a ratio of 1 part to 10 parts of the PDMS by volume and stirred with the metal spatula until combined and well mixed. The mixture is then stored in a glass desiccator connected to a vacuum pump and degassed for 30 min to remove air bubbles in the composite. Next, the mixture is cast into a glass petri dish (D = 90 mm), allowed to settle, and degassed for an additional 10 min to remove remaining air bubbles. The degassed composites are then cured at 100 °C for 35 min for the pristine sample, and 100 °C for 1 h for the composite samples. The cross‐sectional morphology of the composites was characterized by scanning electron microscopy (SEM) images taken using a Hitachi SU8240 at a voltage of 3.0 kV and a current of 5.0 µA.

**Table 1:** Composition of PDMS‐filled composites fabricated for this study.

Sample ID	Filler	Filler mass loading [wt%]	Filler volume loading [vol%]	Composite thickness [mm]
PDMS	None	0	0	0.90
PDMS‐CF	Milled CF	16.3	10	0.90
PDMS‐GP	Graphite Powder	19.2	10	0.90
PDMS‐Cu	Cu Metal Powder	49.1	10	0.90

The parallel plate capacitors were fabricated by cutting a 2 × 2 cm square of the composite and placing it between two pieces of conductive copper tape, as low‐resistance electrodes. The capacitor structure was then connected to a standard Sub‐miniature‐A (SMA) RF Connector by soldering leads of < 1 cm to the signal and ground pins.

The capacitor samples were characterized using a Vector Network Analyzer (VNA) from Pico Technology (PicoVNA 106, covering 300 kHz–6 GHz) connected to a phase‐stable Mini‐Circuits CBL‐1.5M‐SMSM+ cable (L = 1.5 m) and a braided copper cable (L = 15 cm). The VNA was calibrated using a full two‐port Short, Open, Load, Through (SOLT) traceable calibration kit (TA345 SOLT‐STD‐F). The broadband response of each device was measured up to 600 MHz between room temperature (20 °C) to 110 °C, by connecting devices to the VNA and securing them to a hot plate with polyimide tape (Figure S3, Supporting Information).

The capacitance (*C*) of parallel plate capacitors in this work is calculated using Equation (2):

(2) C=−1ImZ·2πfImZ<0

Where Im{*Z*} is the imaginary component of the measured complex impedance, and *f* is the frequency. At lower frequencies, it can be assumed that the parasitic inductance is not significant below the self‐resonant frequency where there is a transition between capacitance being dominant and inductance being dominant.^[^ ^51^ ^]^

### Fabrication and Characterization of Wireless Capacitor Sensors

To achieve wireless sensing, temperature‐sensitive capacitors with coupled inductive coils were integrated. The coils and resulting LC resonant circuits tuned to 6.78 MHz were designed using Keysight ADS resulting in a 9‐turn, 10 × 10 cm square inductor (Figure S15, Supporting Information) and fabricated on polyimide substrates with copper traces, in a standard flexible PCB process. The properties of the coil were tuned using standard high‐*Q* ceramic capacitors (Figure S16, Supporting Information). The resonant frequency (*f*) of the LC network changes in response to capacitance according to Equation (3), where *L* is the inductance and *C* is the capacitance of the circuit.

(3) $$f=\frac{1}{2\pi \sqrt{LC}}$$

The PDMS and PDMS‐CF capacitors were soldered to stranded core wires and connected to an inductive coil in series to form the sensor. The reader coil was tuned to 6.78 MHz with a series capacitor of 66pF. The composite capacitor was placed on a hot plate identical to the one‐port measurement setup and the coils were separated by 45 mm using a piece of Styrofoam. The entire system was then characterized in the range of 0.5–30 MHz in the range of 20–110 °C using the standard VNA, or a portable NanoVNA,^[^ ^66^ ^]^ in the sensing demonstration.

### Fabrication and Characterization of Smart Packaging Demonstrator

A passive sensor tag was fabricated by integrating a PDMS‐CF capacitor with an area of 4 cm^2^ (4 cm length by 1 cm width) into an *RLC* circuit with a 47 Ω resistor and an identical 9‐turn, 10 × 10 cm square inductor coil to those described in Section 2.3. The tag was placed at the bottom of a 6 cm‐tall plastic food container with a gel phantom placed on top to act as a food product. The package was heated with a hot air gun and the reflection coefficient of the reader coil (*S_11_*) was sampled every 1.5 s.

### Humidity Sensing

To measure the responsiveness of PDMS‐CF to humidity, the chipless tag and reader coil were placed inside a sealed cupboard with a humidifier and a Temperature and Humidity Data Logger (RS Pro) mounted next to the tag. The tag was first measured at ambient temperature (20 °C) and humidity (43.5%) for 2 min. The humidifier was then turned on and humidity was allowed to increase in the cupboard until condensation formed on the tag (RH = 93.5%). The cupboard was then evacuated to allow the humidity to return to ambient conditions, and the tag was measured until the condensation had completely evaporated.

### Chipless Temperature Sensing Array Fabrication

A 3 × 2 arrays of passive sensor tags were fabricated by integrating PDMS‐CF capacitors with areas of 0.7, 1, and 4 cm^2^ into RLC circuits using methods described in “Fabrication and Characterization of Smart Packaging Demonstrator” with a 47 Ω resistor and two different coils. The first coil type is a five‐turn circular inductive coil with a total diameter of 5 cm and the second coil type is a five‐turn square inductive coil with a maximum side length of 5 cm. The circular and square coils have trace widths of 0.7 and 1 mm, with separation distances of 1 and 1.5 mm, respectively. The inductance, resistance, and quality factor of the coils are reported in Figure S22 (Supporting Information).

The reader coils for the sensor array were fabricated with a meandering one‐turn coil consisting of a solid‐core wire soldered to an SMA connector. The reader coil was designed to have each sensor coil achieve maximum coupling by overlapping on three edges. Each reader coil was connected to the low‐cost and open‐source VNA (nanoVNA) to yield a pseudo‐two‐port device and take dynamic temperature response measurements in the range of 5–50 MHz. The array was fabricated by affixing sensor coils side‐by‐side in two columns and three rows. The temperature response of the tags in the array was measured using a hot air gun analogous to the procedure described in “Fabrication and Characterization of Smart Packaging Demonstrator.”

## Conflict of Interest

The authors declare no conflict of interest.

## Supporting information



Supporting Information

## Data Availability

The data that support the findings of this study are available in the supplementary material of this article. An open access dataset is hosted at https://doi.org/10.5525/gla.researchdata.1857. [Correction added on 3 February 2025, after first online publication: an open access dataset link is added.]
