# Supplementary material for: Large‐Area Conductor‐Loaded PDMS Flexible Composites for Wireless and Chipless Electromagnetic Multiplexed Temperature Sensors
Source: Adv Sci (Weinh). 2025 Jan 28;12(17):2412066. doi: 10.1002/advs.202412066 (PMC12061244; doi:10.1002/advs.202412066)
Supplement: Supplementary file 1 — Supporting Information [file ADVS-12-2412066-s001.pdf]

ADVANCED SCIENCE
Open Access

Supporting Information

for *Adv. Sci.*, DOI 10.1002/advs.202412066

Large-Area Conductor-Loaded PDMS Flexible Composites for Wireless and Chipless Electromagnetic Multiplexed Temperature Sensors

*Benjamin King, Nikolas Bruce and Mahmoud Wagih**

Large-Area Conductor-Loaded PDMS Flexible Composites for Wireless and Chipless Electromagnetic Multiplexed Temperature Sensors

Benjamin King, Nikolas Bruce, and Mahmoud Wagih*

University of Glasgow, James Watt School of Engineering, Glasgow, UK

mahmoud.wagih@glasgow.ac.uk

Supporting Information

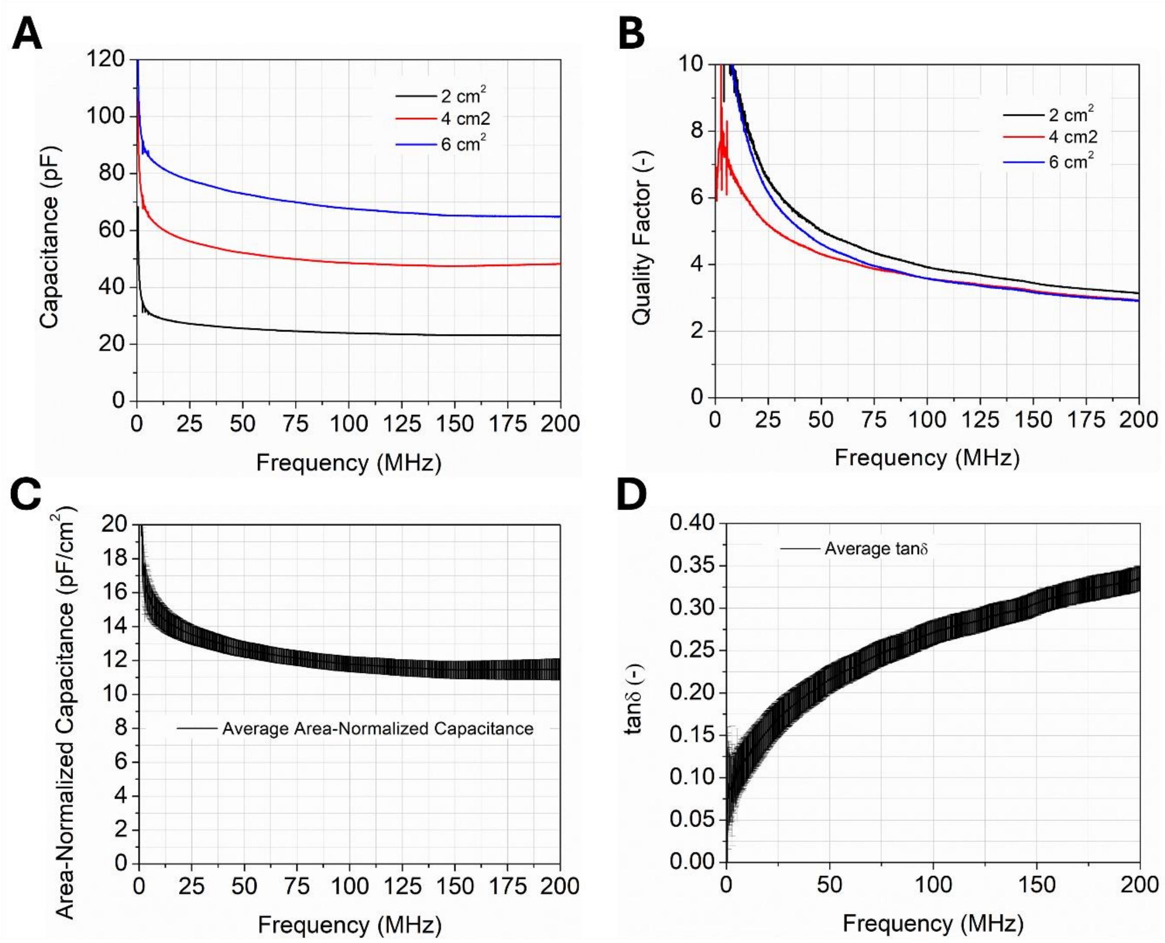

Figure S1. RF characterization of the sensing capacitors and their scalability: A) Capacitance and B) quality factor for PDMS-CF capacitors with area of 2 cm^2 – 6 cm^2 from 1 MHz to 200 MHz. C) area-normalized capacitance and D) area-normalized loss tangent (tanδ) of PDMS-CF capacitors with area of 2 cm^2 – 6 cm^2. Error bars in Figures C and D are for n = 3 area-normalized samples.

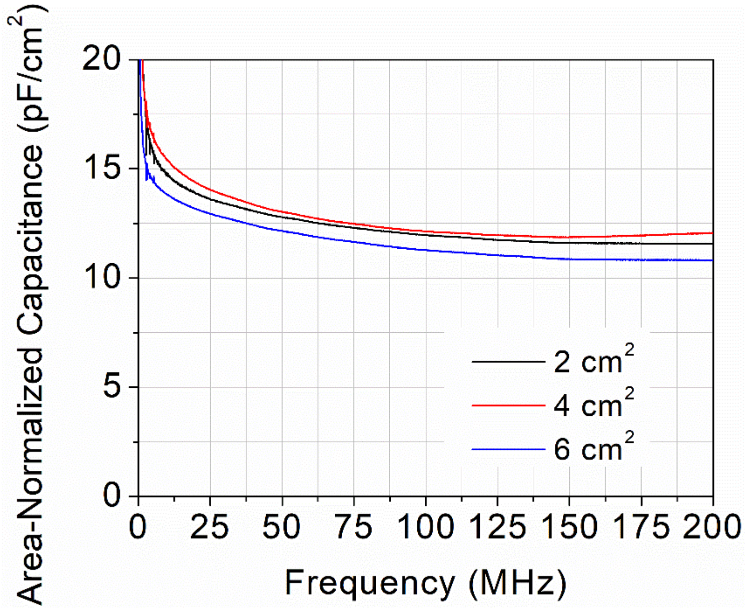

Figure S2. Area-normalized capacitance for PDMS-CF capacitors of 2 cm^2 (black line), 4 cm^2 (red line) and 6 cm^2 (blue line) from 10 MHz to 200 MHz.

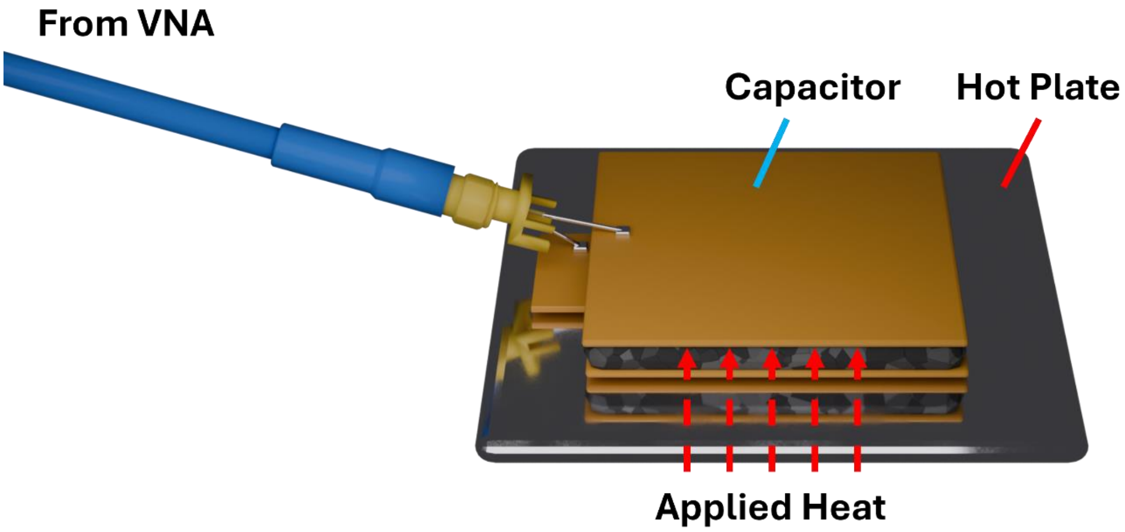

Figure S3. Schematic of PDMS composite capacitor temperature sensing work bench.

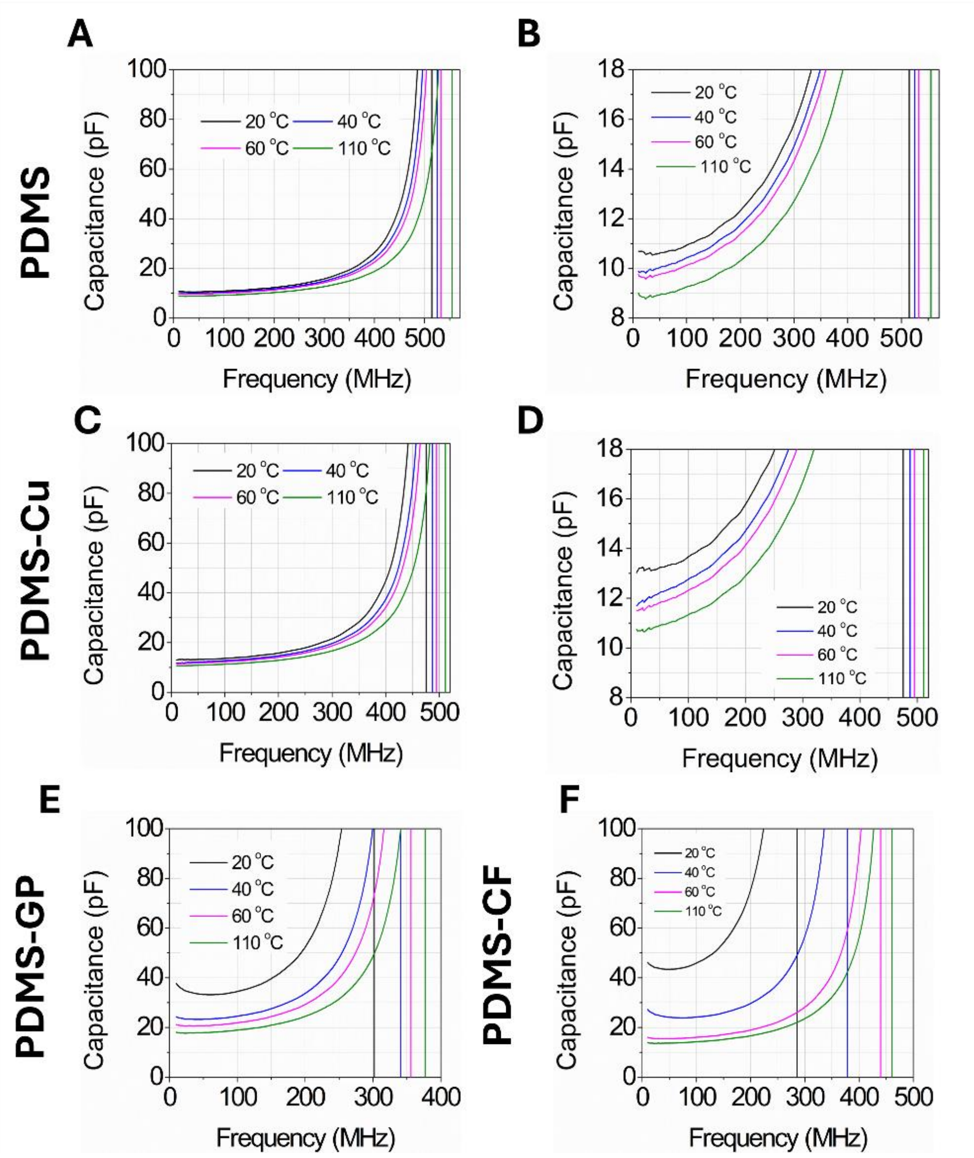

Figure S4. Broadband capacitance data up to the self-resonant frequency for conductive composites at 20 °C, 40 °C, 60 °C and 110 °C for A and B) PDMS C and D) PDMS-Cu, E) PDMS-GP and F) PDMS-CF parallel plate capacitors with an area of 4 cm^2. B and D are insets of A and C, respectively.

Table S1. Mean sensing response and sensitivity for loaded composite capacitors.

Sample ID	Filler	S x10^{-2} at 10 MHz (MHz·°C^{-1})	S x10^{-2} at 100 MHz (MHz·°C^{-1})	S x10^{-2} at 200 MHz (MHz·°C^{-1})
Pristine	None	0.14	0.16	0.18
PDMS-CF*	Milled CF	1.99	2.17	2.35
PDMS-GP*	Graphite Powder	1.31	1.08	1.39
PDMS-Cu	Cu Metal Powder	0.16	0.18	0.19

*Sensitivity data taken from 20 °C to 50 °C.

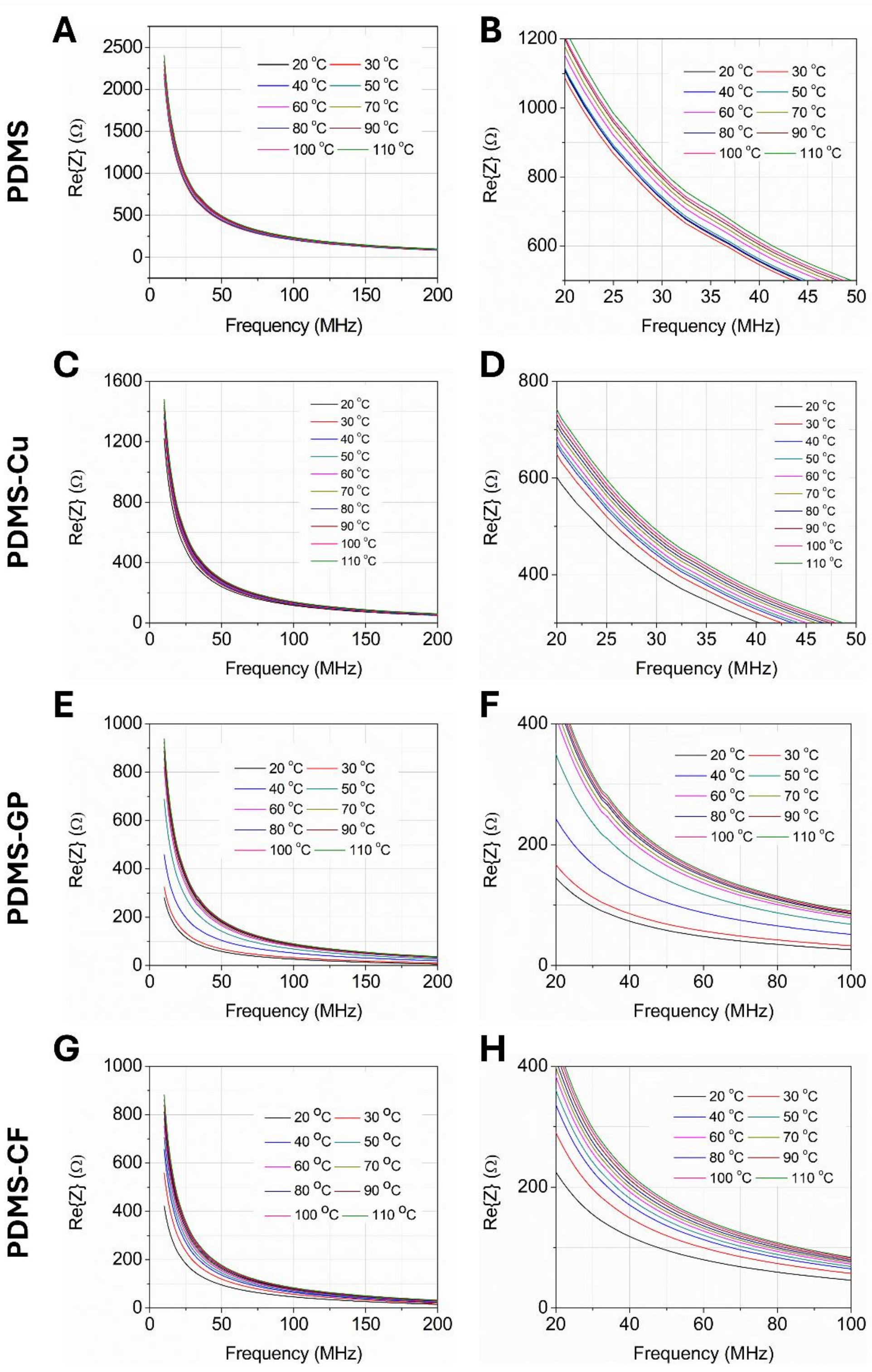

Figure S5. Broadband real impedance data up to 200 MHz (B, D, F, and H are an inset of A, C, E and G, respectively) for A and B) PDMS C and D) PDMS-Cu, E and F) PDMS-GP and G and H) PDMS-CF parallel plate capacitors with an area of 4 cm^2.

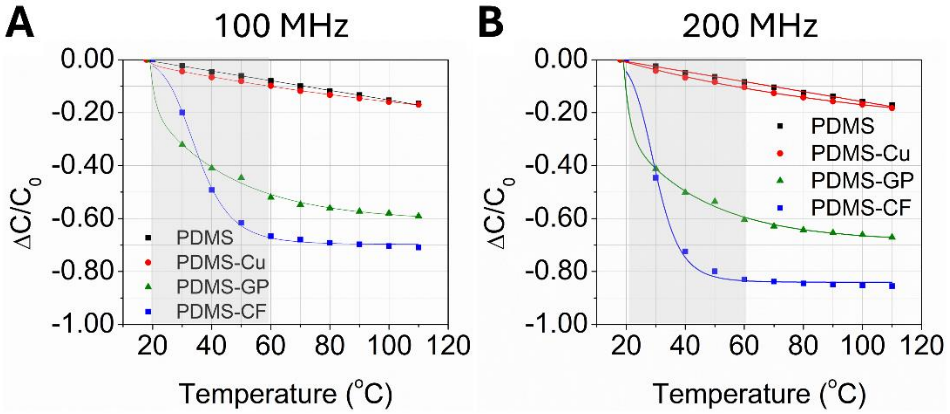

Figure S6. Frequency-dependant relative response to temperature of loaded PDMS capacitors at A) 100 MHz and B) 200 MHz. The reference temperature for these measurements was taken at room temperature (20 °C) and the shaded region is the target sensing range. Measurements were taken for composite capacitors of pristine PDMS (black squares), PDMS-Cu (red circles), PDMS-GP (green triangles) and PDMS-CF (blue squares).

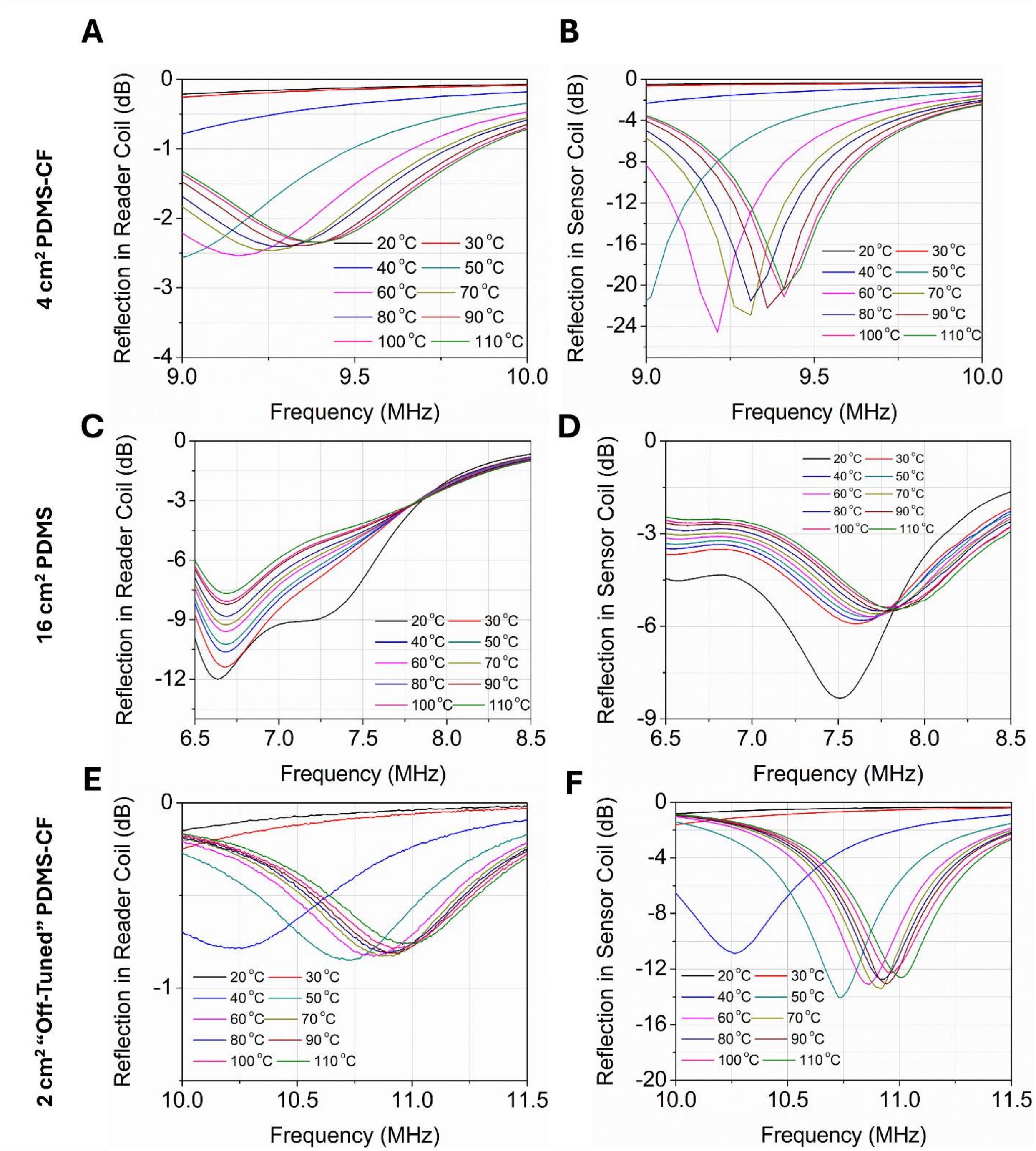

Figure S7. Inset of reflection coefficient (S_{11}) data for tuned reader coils (A, C, and E) and sensor coils (B, D, and F) from Figure 3. A and B) 4 cm^2 PDMS-CF capacitor. C and D) 16 cm^2 tuned PDMS capacitor. E and F) Detuned 2 cm^2 PDMS-CF capacitor with a reference resonant frequency of 8.45 MHz.

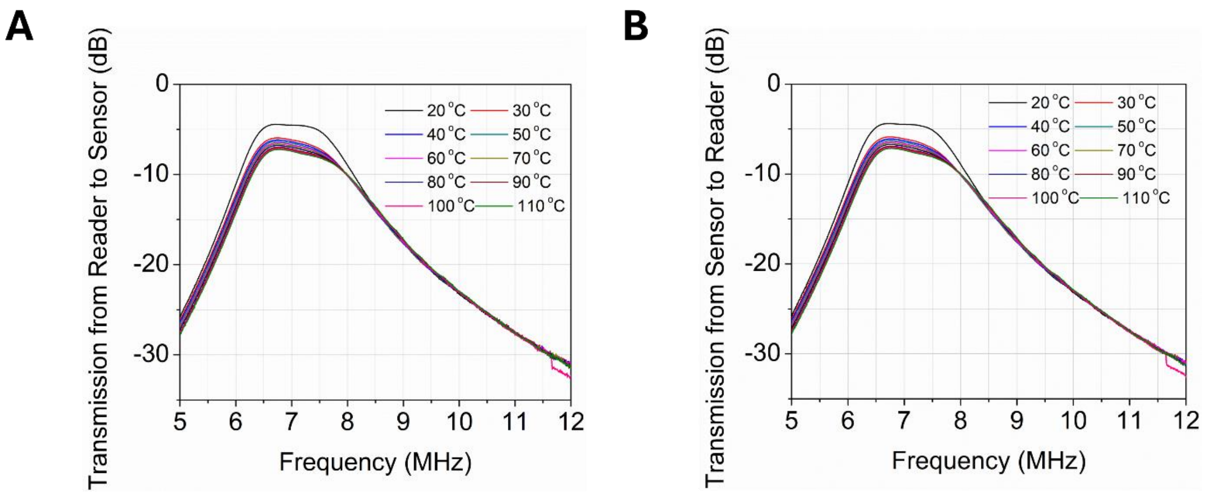

Figure S8. The PDMS sensor's S21 response, indicating minimal amplitude or frequency variations A) S12 transmission coefficient and B) S21 transmission coefficient data for 16 cm² active area wireless PDMS capacitor sensor with a distance between coils of 45 mm.

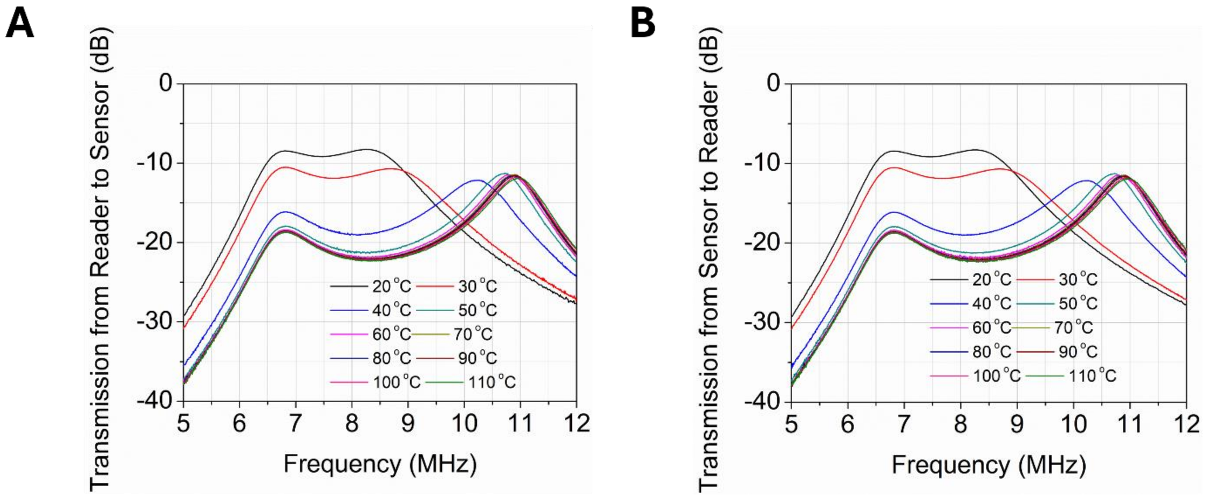

Figure S9. The 2 cm² PDMS-CF sensor's transmission response A) S12 transmission coefficient and B) S21 transmission coefficient data for wireless 2cm² active area PDMS-CF capacitor sensor with a distance between coils of 45 mm.

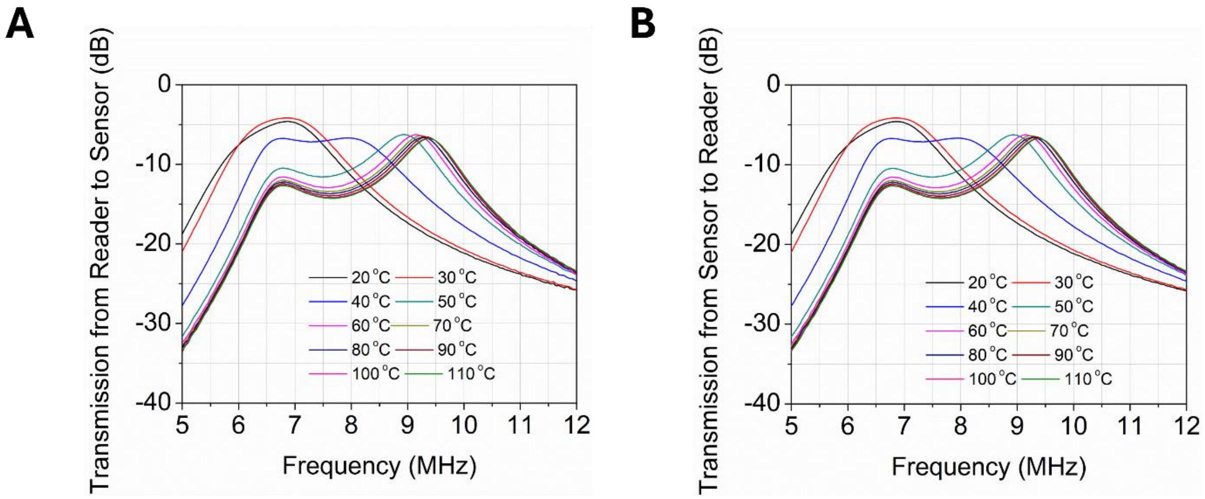

Figure S10. The 4 cm² PDMS-CF sensor's transmission response A) S12 transmission coefficient and B) S21 transmission coefficient data for 4cm² active area PDMS-CF capacitor sensor with a distance between coils of 45 mm.

Table S2. Comparison with previously reported frequency-domain and AC/RF temperature sensors.

Reference	Sensor Architecture	Active Material	Temperature Sensing Range ΔT (°C)	Centre Frequency, f_0 (MHz)	Sesitivity (MHz·°C^{-1})	Frequency-Normalized Sensitivity (%·°C^{-1})
[S1]	Dielectric resonator (chipless tag)	Ceramic	20 – 370	2920	0.307	0.0102
[S2]	Chipless split-ring resonator	Ceramic	28 - 1100	2470	0.0956	0.00395
[S3]	Near-field LC resonator	Ceramic	19 – 900	36	0.00522	0.0158
[S4]	Dual split-ring resonator	Ceramic	23 – 200	11930	0.462	0.00389
[S5]	Dual split-ring resonator	Ceramic	25 – 135	2030	0.068	0.0034
[S6]	Patch antenna	Polymer	33 – 77	5000	0.205	0.0041
[S7]	Microstrip resonator	Rogers 3210	30 – 80	2400	0.5	0.0208
[S8]	Bulk acoustic wave resonator	Ceramic	10 – 80	2480	0.1	0.00403
[S9]	LC Resonator	Ceramic	500 – 1200	54.5	0.00357	0.00655
[S10]	Cylindrical antenna / resonator	Ceramic	0 – 400	10500	0.35	0.00333
[S11]	RF cavity	Metal	20 – 170	2493	0.005	0.006
[S12]	Patch antenna	Conductive polymer composite	35 – 205	2000	3.17	0.124
	Resonator 2		35 – 205	1770	3.29	0.186
	Resonator 1		35 – 110	820	2.14	0.262
[S13]	Log-periodic dipole antenna (LPDA) and cavity filter	Ceramic	20 – 1400	54.5	0.356	0.032
[S14]	3D photonic crystal	Ceramic	0 – 1400	84000	5	0.0714
This work	Parallel plate capacitor and inductive coils	Conductive polymer composite	20 – 110	6.8-7.0	0.028-0.036	0.38 – 0.55

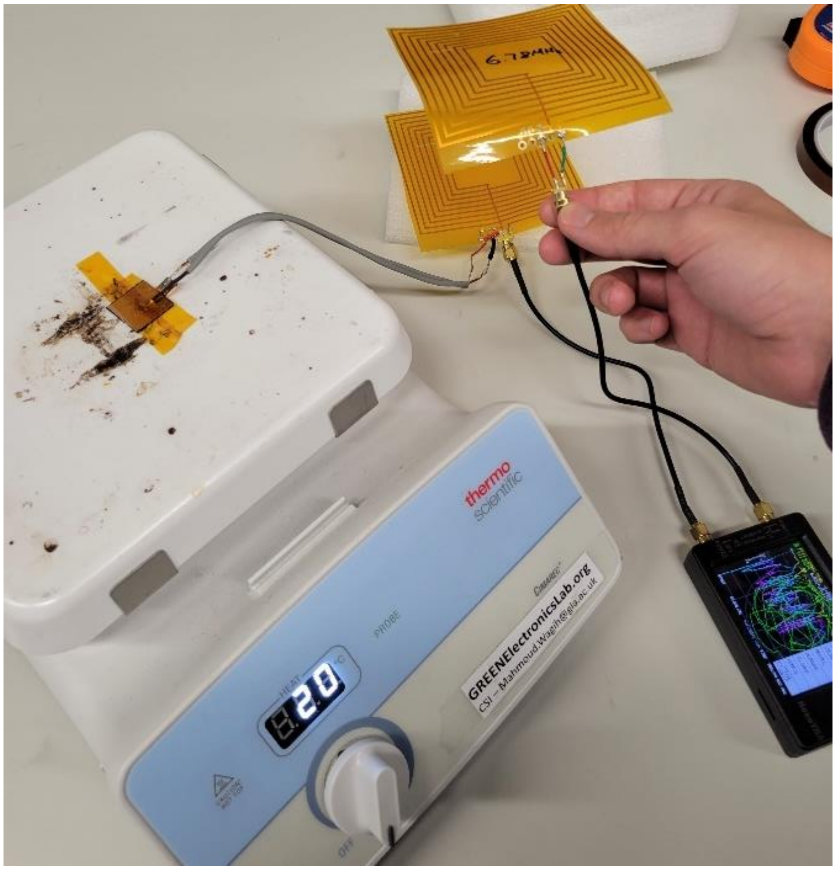

Figure S11. Photograph of NanoVNA wireless temperature sensing setup.

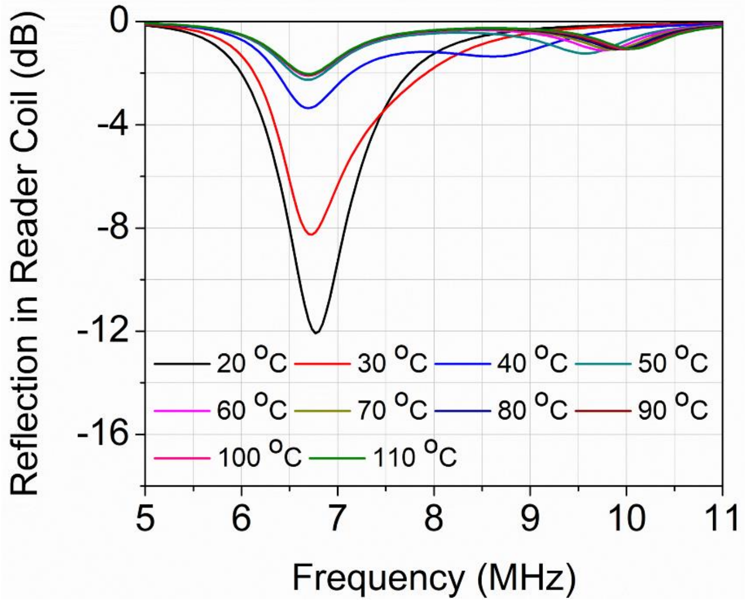

Figure S12. Reflection coefficient (S_{11}) data of 4 cm^2 wireless capacitor read by handheld (Nano) VNA.

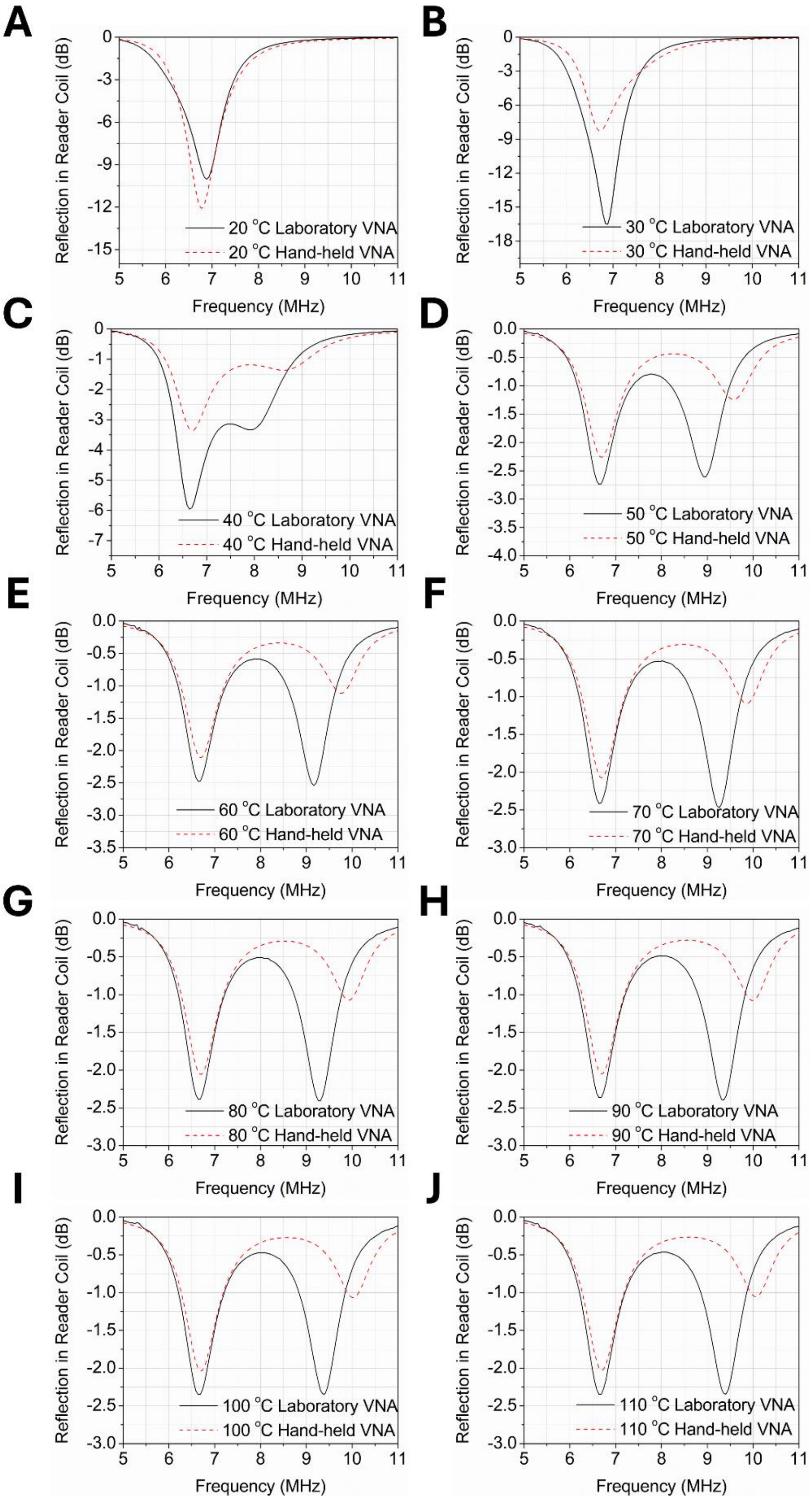

Figure S13. Reflection coefficient (S_{11}) data from 20°C - 110°C for PDMS-CF measured with the laboratory-grade VNA (solid lines) and hand-held VNA (dashed line).

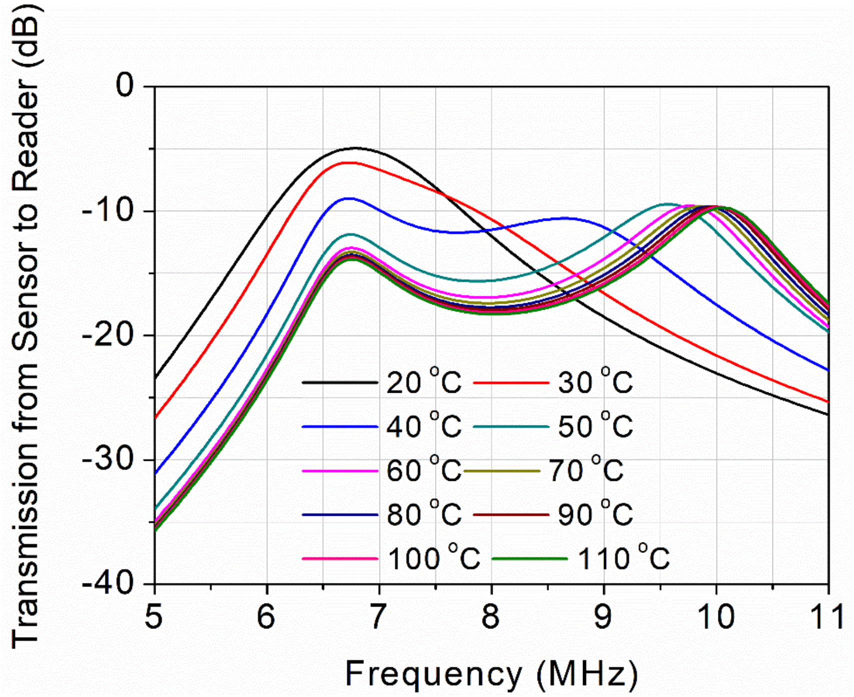

Figure S14. Transmission coefficient (S_{21}) data from 20°C - 110°C for PDMS-CF measured with the NanoVNA.

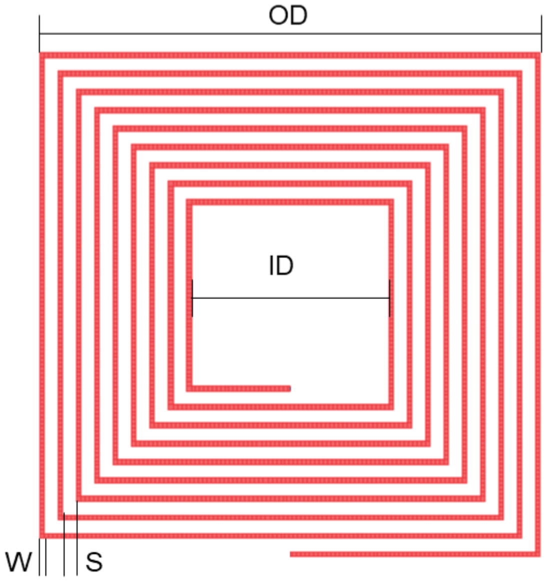

Parameter	Description	Measurement (mm)
OD	Outer Diameter	100
ID	Inner Diameter	39.36
W	Width of Trace	1
S	Conductor Spacing	2.665
N	Number of Turns	9

Figure S15. Layout and dimensions of the inductive coils used in this work.

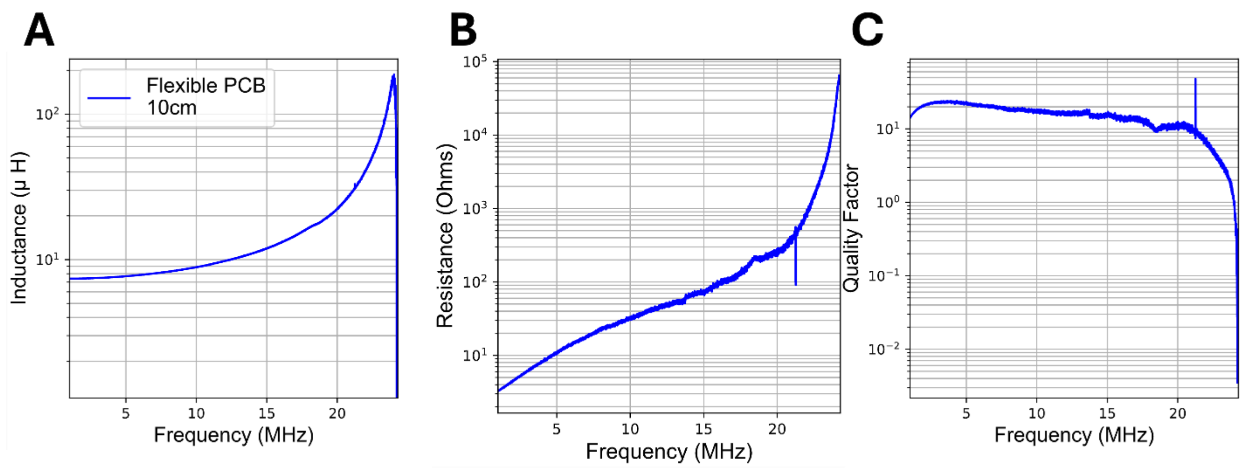

FigureS16. Properties of 10 cm square inductive coils employed in the wireless chipless sensor interface. A) Measured Inductance, B) Measured resistance, and C) measured capacitance. The reader coil was tuned using standard high-Q ceramic capacitors

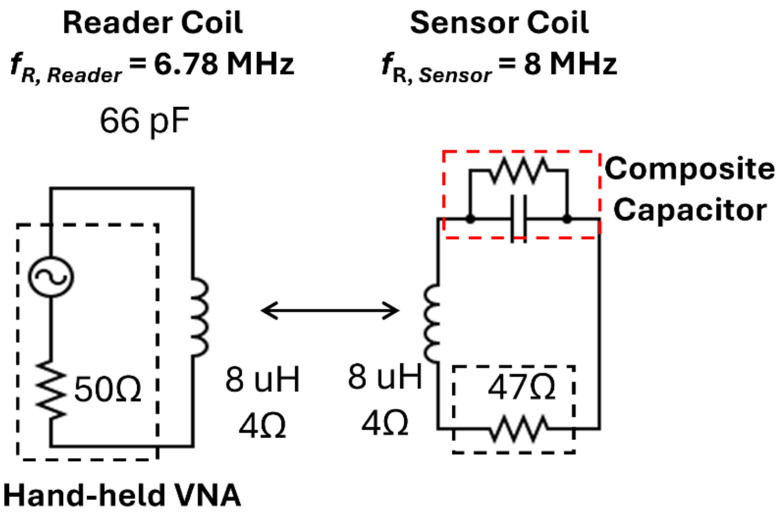

Figure S17. Equivalent circuit of unloaded resonant frequency of reader coil and sensing coil with 4 cm^2 PDMS-CF capacitor and 47 Ω resistor in the sensor coil.

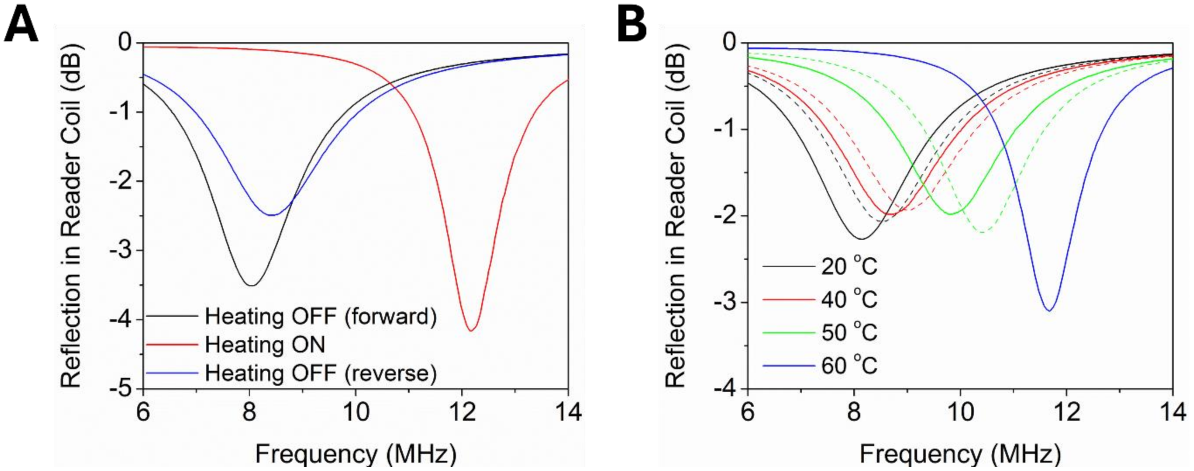

Figure S18. Broadband response of sensor tag with 47 Ω resistor and 4 cm^2 PDMS-CF capacitor in parallel with inductive coil and RL reader coil. A) Reflection coefficient (S_{11}) before heating (black line), at steady state with applied heat (red line) and with heating removed (blue line). B) Reflection coefficient (S_{11}) at different temperature steps from 20 °C to 60 °C with forward steps (solid lines) and reverse steps dashed line).

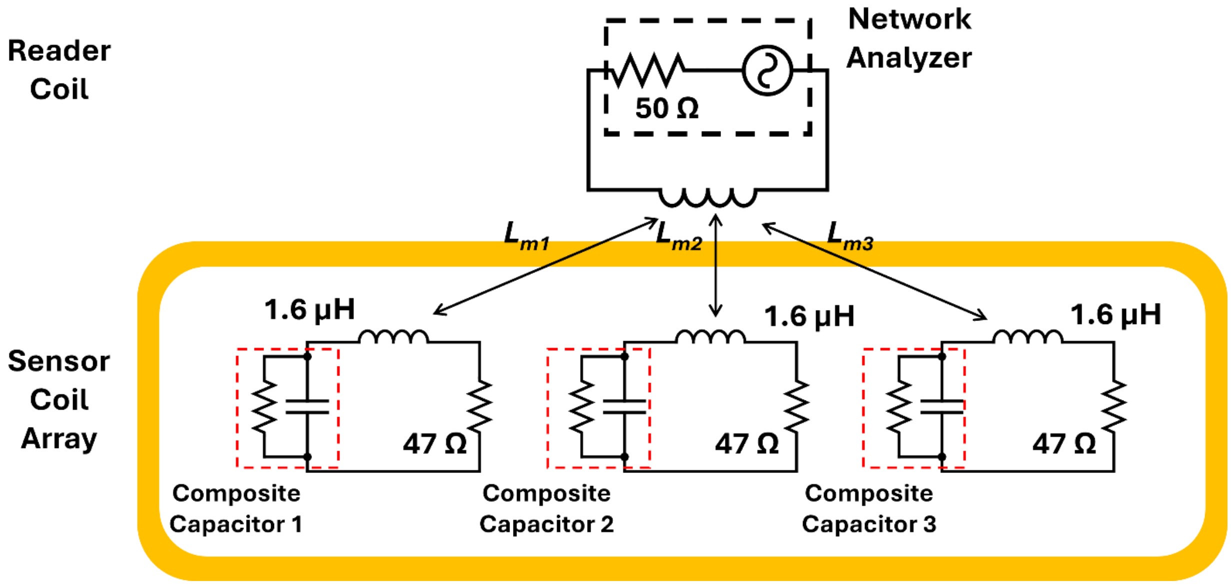

Figure S19. Circuit Diagram of chipless temperature sensing array. *L_i represents mutual inductance between each sensor coil and the reader coil.*

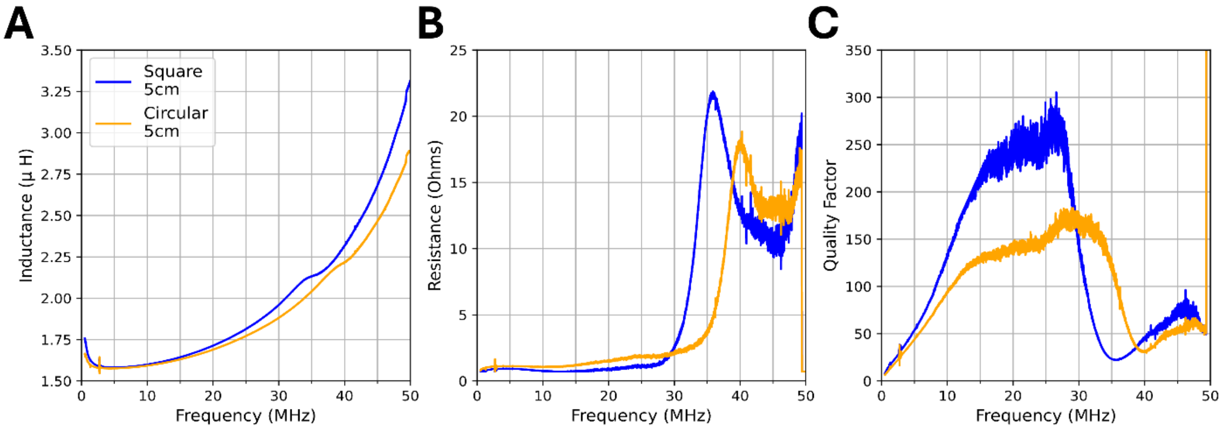

Figure S20. Properties of 5 cm square (blue lines) and circular (orange lines) inductive sensor coils in temperature sensor array. A) Measured inductance, B) Measured resistance, and C) measured capacitance. The coils were tuned using standard high-Q ceramic capacitors.

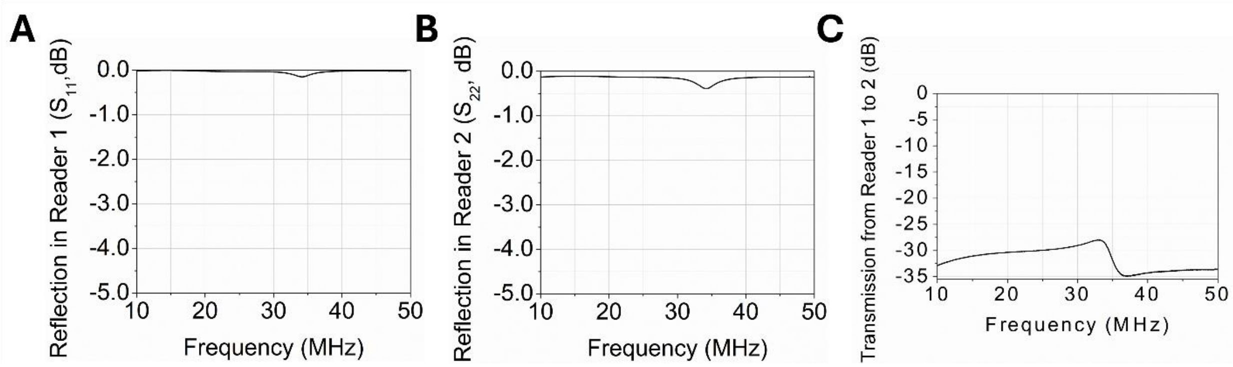

Figure S21. Broadband data for mated one-turn wire reader coils. A) Reflection coefficient (S_{11}) for reader 1, B) S_{22} for reader 1, and C) S_{12} between the readers.

[S1] Kubina, B.; Schusler, M.; Mandel, C.; Mehmood, A.; Jakoby, R. Wireless High-Temperature Sensing with a Chipless Tag Based on a Dielectric Resonator Antenna. *Proc. IEEE Sensors* **2013**, p. 1-4. DOI: 10.1109/ICSENS.2013.6688181.

[S2] Lu, F.; Tan, Q.; Ji, Y.; Guo, Q.; Guo, Y.; Xiong, J. A Novel Metamaterial Inspired High-Temperature Microwave Sensor in Harsh Environments. *Sensors* **2018**, *18* (9), 2879. DOI: 10.3390/s18092879.

[S3] Tan, Q.; Ren, Z.; Cai, T.; Li, C.; Zheng, T.; Li, S.; Xiong, J. Wireless Passive Temperature Sensor Realized on Multilayer HTCC Tapes for Harsh Environment. *J. Sensors* **2015**, *2015, 124058*. DOI: 10.1155/2015/124058.

[S4] Karim, H.; Delfin, D.; Chavez, L. A.; Delfin, L.; Martinez, R.; Avila, J.; Rodriguez, C.; Rumpf, R. C.; Love, N.; Lin, Y. Metamaterial Based Passive Wireless Temperature Sensor. *Adv. Eng. Mater.* **2017**, *19* (5), 1600741. DOI: 10.1002/adem.201600741.

[S5] Wang, C.; Chen, L.; Tian, B.; Jiang, Z. High-Linearity Wireless Passive Temperature Sensor Based on Metamaterial Structure with Rotation-Insensitive Distance-Based Warning Ability. *Nanomaterials* **2023**, *13* (17), 2482. DOI: 10.3390/nano13172482.

[S6] Tchafa, F. M.; Huang, H. Microstrip Patch Antenna for Simultaneous Temperature Sensing and Superstrate Characterization. *Smart Mater. Struct.* **2019**, *28* (10), 065019. DOI: 10.1088/1361-665X/ab2213.

[S7] Leier, B.; Baghelani, M.; Iyer, A. K. A Microwave Stripline Ring Resonator Sensor Exploiting the Thermal Coefficient of Dielectric Constant for High-Temperature Sensing. *IEEE Sens. J.* **2022**, *22* (22), 21666–21675. DOI: 10.1109/JSEN.2022.3210779.

[S8] Lin, J. H.; Kao, Y. H. Wireless Temperature Sensing Using a Passive RFID Tag with Film Bulk Acoustic Resonator. *Proc. - IEEE Ultrason. Symp.* **2008**, 2209–2212. DOI: 10.1109/ULTSYM.2008.0547.

[S9] Idhaiam, K. S. V.; Pozo, P. D.; Sabolsky, K.; Sabolsky, E. M.; Sierros, K. A.; Reynolds, D. S. All-Ceramic LC Resonator for Chipless Temperature Sensing within High Temperature Systems. *IEEE Sens. J.* **2021**, *21* (18), 19771–19779. DOI: 10.1109/JSEN.2021.3094406.

[S10] Cheng, H.; Ren, X.; Ebadi, S.; Chen, Y.; An, L.; Gong, X. Wireless Passive Temperature Sensors Using Integrated Cylindrical Resonator/Antenna for Harsh-Environment Applications. *IEEE Sens. J.* **2015**, *15* (3), 1453–1462. DOI: 10.1109/JSEN.2014.2363426.

[S11] Ghafourian, M.; Bridges, G. E.; Nezhad, A. Z.; Thomson, D. J. Wireless Overhead Line Temperature Sensor Based on RF Cavity Resonance. *Smart Mater. Struct.* **2013**, *22* (7), 075010. DOI: 10.1088/0964-1726/22/7/075010.

[S12] Wagih, M.; Shi, J.; Li, M.; Komolafe, A.; Whittaker, T.; Schneider, J.; Kumar, S.; Whittow, W.; Beeby, S. Wide-Range Soft Anisotropic Thermistor with a Direct

Wireless Radio Frequency Interface. *Nat. Commun.* **2024**, *15* (1), 452. DOI: 10.1038/s41467-024-44735-z.

[S13] Peng, H.; Yang, X.; Wu, X.; Peng, W. A Wireless Temperature Sensor Applied to Monitor and Measure the High Temperature of Industrial Devices. *IEEE Trans. Antennas Propag.* **2024**, *72* (6), 5273–5282. DOI: 10.1109/TAP.2024.3389213.

[S14] Sánchez-pastor, J.; Jakoby, R.; Benson, N.; Jiménez-sáez, A.; Benson, N.; Jim, A. A wireless W-band 3D-printed temperature sensor operating beyond 1000 °C. *Preprint* **2023**. DOI: 10.21203/rs.3.rs-3394921/v1.
